# Supplementary material for: Agreement between self-reported and researcher-measured height, weight and blood pressure measurements for online prescription of the combined oral contraceptive pill: an observational study
Source: BMJ Open. 2022 May 24;12(5):e054981. doi: 10.1136/bmjopen-2021-054981 (PMC9131065; doi:10.1136/bmjopen-2021-054981)
Supplement: Supplementary data [file bmjopen-2021-054981supp001.pdf]

Supplementary material

A. Process of BP measurement and examples from free text answers

Process of blood pressure measurement	Example
Unclassifiable	- I have a blood pressure monitor at home (date given between order and research visit)<br>- "My blood pressure was measured by a colleague at work (12 days before order)<br>- "[South East London Clinic] (date given between order and research visit)"<br>- "I have a nurse at my office that offer blood pressure testing so I used the facilities provided to check (date given between order and research visit)"<br>- "I visited the pharmacy stand in boots. They took me to a private room and took my blood pressure for me (date given between order and research visit)"
Clinically appropriate	- "From my GP when I was first prescribed the pill (3 months before order)"<br>- "The nurse in the clinic took my blood pressure (same day as order)"<br>- "Inherited blood pressure machine from relative with long term illness who no longer needed it. Used this at home to obtain measure (4 days before order)<br>- "I visited my aunts house to get my blood pressure reading since she has a machine at home (day before order)<br>- "I went to my local pharmacy where they had a self checker machine that calculated my blood pressure, weight and height (same day as order)"
Clinically inappropriate	- "At the GP before getting a repeat prescription of the pill, which was measured normally (18 months before order)"<br>- "A bupa nurse came into my place of work and offered a health check. My blood pressure was taken there (2 years before order)<br>- "I looked up what a healthy blood pressure would be on NHS website and put in that blood pressure when on the sh:24 website"<br>- "Estimated. Had a blood pressure machine but feel I did not use it correctly"<br>- "Googled healthy blood pressure"

McCulloch H, *et al. BMJ Open* 2022; 12:e054981. doi: 10.1136/bmjopen-2021-054981

B. Self-reported and researcher-measured BMI

		Measured BMI		
		**UKMEC 1&2<br><35.0 kg/m**	**UKMEC 3&4<br>> or= 35 kg/m**	**Total**
**Self-reported BMI**	**UKMEC 1&2<br><35.0 kg/m**	358	1	359
	**UKMEC 3&4<br>> or= 35 kg/m**	2	4	6
	**Total**	360	5	365

C. Self-reported and researcher-measured BP

		Researcher-measured blood pressure (mmHg)		
		**< 140/90**	**> or = 140/90**	**Total**
**Self-reported blood pressure (mmHg)**	**<140/90**	344	10	354
	**> or= 140/90**	10	1	11
	**Total**	354	11	365

D. SMS invitation message

“Hello. SH:24 are working with King's College Hospital to improve contraception services. King's would like to invite you to take part in their research - they will thank you for your time with £45. The research would involve meeting a member of the King's team to allow them to check your height, weight and blood pressure. You will also be asked to complete a short questionnaire (this should take no more than 40 mins in total). Text back NO if you don't want to receive a call back from the King's research team. Thanks, SH:24"

McCulloch H, *et al. BMJ Open* 2022; 12:e054981. doi: 10.1136/bmjopen-2021-054981
