# Supplementary material for: Agreement between self-reported and researcher-measured height, weight and blood pressure measurements for online prescription of the combined oral contraceptive pill: an observational study
Source: BMJ Open. 2022 May 24;12(5):e054981. doi: 10.1136/bmjopen-2021-054981 (PMC9131065; doi:10.1136/bmjopen-2021-054981)
Supplement: Author's manuscript [file bmjopen-2021-054981.draft_revisions.pdf]

BMJ Open is committed to open peer review. As part of this commitment we make the peer review history of every article we publish publicly available.

When an article is published we post the peer reviewers' comments and the authors' responses online. We also post the versions of the paper that were used during peer review. These are the versions that the peer review comments apply to.

The versions of the paper that follow are the versions that were submitted during the peer review process. They are not the versions of record or the final published versions. They should not be cited or distributed as the published version of this manuscript.

BMJ Open is an open access journal and the full, final, typeset and author-corrected version of record of the manuscript is available on our site with no access controls, subscription charges or pay-per-view fees (http://bmjopen.bmj.com).

If you have any questions on BMJ Open's open peer review process please email info.bmjopen@bmj.com

BMJ Open

Agreement between self-reported and researcher-measured height, weight and blood pressure measurements for online prescription of the combined oral contraceptive pill: an observational study

Journal:	*BMJ Open*
Manuscript ID	bmjopen-2021-054981
Article Type:	Original research
Date Submitted by the Author:	30-Jun-2021
Complete List of Authors:	McCulloch, Hannah; King's College London Faculty of Life Sciences and Medicine, King's Centre for Global Health and Health Partnerships<br>Morelli, Alessandra; King's College London Faculty of Life Sciences and Medicine, King's Centre for Global Health and Health Partnerships<br>Free, Caroline; London School of Hygiene and Tropical Medicine Faculty of Epidemiology and Population Health, Department of Population Health<br>Syred, Jonathan; King's College London Faculty of Life Sciences and Medicine, King's Centre for Global Health and Health Partnerships<br>Botelle, Riley; King's College London Faculty of Life Sciences and Medicine, Kings Centre for Global Health and Health Partnerships<br>Baraitser, Paula; King's College Hospital, Sexual Health; SH:24 CIC
Keywords:	SEXUAL MEDICINE, REPRODUCTIVE MEDICINE, PUBLIC HEALTH

SCHOLARONE™
Manuscripts

I, the Submitting Author has the right to grant and does grant on behalf of all authors of the Work (as defined in the below author licence), an exclusive licence and/or a non-exclusive licence for contributions from authors who are: i) UK Crown employees; ii) where BMJ has agreed a CC-BY licence shall apply, and/or iii) in accordance with the terms applicable for US Federal Government officers or employees acting as part of their official duties; on a worldwide, perpetual, irrevocable, royalty-free basis to BMJ Publishing Group Ltd ("BMJ") its licensees and where the relevant Journal is co-owned by BMJ to the co-owners of the Journal, to publish the Work in this journal and any other BMJ products and to exploit all rights, as set out in our licence.

The Submitting Author accepts and understands that any supply made under these terms is made by BMJ to the Submitting Author unless you are acting as an employee on behalf of your employer or a postgraduate student of an affiliated institution which is paying any applicable article publishing charge ("APC") for Open Access articles. Where the Submitting Author wishes to make the Work available on an Open Access basis (and intends to pay the relevant APC), the terms of reuse of such Open Access shall be governed by a Creative Commons licence – details of these licences and which Creative Commons licence will apply to this Work are set out in our licence referred to above.

Other than as permitted in any relevant BMJ Author's Self Archiving Policies, I confirm this Work has not been accepted for publication elsewhere, is not being considered for publication elsewhere and does not duplicate material already published. I confirm all authors consent to publication of this Work and authorise the granting of this licence.

For peer review only

Agreement between self-reported and researcher-measured height, weight and blood pressure measurements for online prescription of the combined oral contraceptive pill: an observational study

Hannah McCulloch[1], Alessandra Morelli[1], Caroline Free[2], Jonathan Syred[1], Riley Botelle[1], Paula Baraitser[134]

Author affiliations

[1] King's College London, King's Centre for Global Health and Health Partnerships, London, United Kingdom

[2] London School of Hygiene and Tropical Medicine, Department of Population Health, London, United Kingdom

[3] King's College Hospital, Camberwell Sexual Health Clinic, 94-104 Denmark Hill, SE5 9RS, London, United Kingdom

[4] SH:24, 35A Westminster Bridge Road, SE1 7JB, London, United Kingdom

Corresponding author:

Hannah McCulloch, King's College London, King's Centre for Global Health and Health Partnerships, Weston Education Centre, 10 Cutcombe Rd, SE5 9RJ London, United Kingdom; hrmcculloch@gmail.com

Title word count: 23

Abstract word count: 294

Strengths and limitations statement: 181

Full article word count: 2729

For peer review only

For peer review only - http://bmjopen.bmj.com/site/about/guidelines.xhtml

Abstract

Title: Agreement between self-reported and researcher-measured height, weight and blood pressure measurements for online prescription of the combined oral contraceptive pill: an observational study

Objective(s): To compare agreement between self-reported height, weight and blood pressure measurements submitted to an online contraceptive service with researcher-measured values and document strategies used for self-reporting.

Design: An observational study.

Setting: An online sexual health service which provided the combined oral contraceptive pill, free of charge, to users in Southeast London, England.

Participants: Between August 2017 and August 2019, 365 participants were recruited. Median time between online order and research visit, where biometric measurements were taken, was 20 days (Interquartile range: 14-30).

Primary and secondary outcome measures: The primary outcome, for which the study was powered, was the agreement between self-reported and researcher-measured body mass index and blood pressure measurements, compared using kappa coefficients. Secondary measures of agreement included sensitivity, specificity, and Bland Altman plots. The study also describes strategies used for self-reporting and classifies their clinical appropriateness.

Results: 327 participants fully described their process of blood pressure measurement with 296 (90.5%) classified as clinically appropriate. Agreement between self-reported and researcher-measured body mass index was substantial (0.72 [95% confidence intervals [CI] 0.42, 1.0]), but poor for blood pressure (0.06 [95% CI -0.11, 0.23]). Self-reported height and weight readings identified 80.0% (95% CI 28.4 - 99.5) of individuals with a researcher-measured high body mass index (≥ than 35kg/m2) and 9.1% (95% CI 0.23, 41.3) of participants with a researcher-measured high blood pressure (≥ 140/90 mmHg).

Conclusion: In this study, self-reported metrics were good predictors of researcher-measured body mass index but not blood pressure. This may be due to the inherent variability of blood pressure, overdiagnosis of hypertension by researchers due to ‘white coat syndrome’ or inaccurate self-reporting. As online contraceptive services are increasingly prevalent and self-measured blood pressure is accurate in other populations, strategies to improve self-reporting of blood pressure for remote prescription of the combined pill are needed.

For peer review only

For peer review only - http://bmjopen.bmj.com/site/about/guidelines.xhtml

Strengths and limitations of this study

- This is the first study of its kind to observe self-reporting of blood pressure and body mass index to obtain the combined oral contraceptive pill in a young, healthy population of reproductive age.
- The study is timely as innovative, self-care interventions for sexual and reproductive health become more prevalent.
- Although, prior to contraception provision, clinicians taking single measures of blood pressure is standard practice, the assumption that the measurement from the research visit reflects the true blood pressure is problematic due to the natural variation of blood pressure.
- Whilst researchers employed recommended strategies to mitigate inaccuracies in blood pressure measurement, some factors remain irremediable, for example 'white coat syndrome', whereby researcher-, or clinician-, measured blood pressure is artificially raised due to anxiety, or natural blood pressure variation.
- We recognise the potential for self-selection bias in recruitment, where users who may have estimated their blood pressure during their order might have been unwilling to participate.

Key words: Online sexual health services, contraception, self-care, self-reported biometric data, remote prescribing, blood pressure

For peer review only - http://bmjopen.bmj.com/site/about/guidelines.xhtml

For peer review only

Full article

1. Introduction

In the United Kingdom (UK), 29.5% of sexually active women of reproductive age use oral contraceptives [1]. Whilst contraception is available free of cost in the UK, research has found that 37.0% of women have difficulty accessing contraceptive services [2].

Online sexual health services, offering tests for sexually transmitted infections (STIs) ordered online and posted home, with self-taken samples posted to laboratories and results communicated remotely, have become part of the UK sexual health economy. Online services increase access to testing, including amongst those who have never tested before [3, 4].

Online services might facilitate access to contraception, enabling users to order oral contraception with remote prescribing and postal delivery [5]. Remote prescription of the combined oral contraceptive (COC) requires a medical history and self-reported height, weight and blood pressure (BP). The accuracy of these biometric measurements is important to ensure appropriate, safe prescription.

The UK and World Health Organisation (WHO) medical eligibility criteria (UKMEC/WHOMEC) classify all contraceptive contraindications on a scale of 1-4 [6, 7]. A classification of 1 or 2 implies that benefits of COC use outweigh the risks, whilst a classification of 3 or 4 suggests that risks of use outweigh benefits. A body mass index (BMI) equal to or greater than 35kg/m^2 or a BP consistently above 140/90 mmHg would be classified as UKMEC 3 or 4.

We studied a UK-based, online contraceptive service, SH:24, which provided COC via post after an online medical history and self-reported height, weight and BP [8]. We aimed to compare self-reported BMI and BP measurements with measurements taken by researchers.

2. Materials and methods

2.1 Setting and ethical approval

SH:24 provided online sexual health services free to the residents of five areas of South East London, areas with poor sexual and reproductive health (SRH) indicators [9, 10], between January 2017 and August 2019. SH:24 is an NHS-commissioned-service, registered with the UK Quality Care Commission and compliant with the General Medical Council guidance on remote prescribing [11]. SH:24 was advertised through online media, and signposting from clinics. The study was given ethical approval by the East Midlands – Leicester Research Ethics Committee on 6th June 2017.

2.2 Data collection

For peer review only

For peer review only - http://bmjopen.bmj.com/site/about/guidelines.xhtml

Users who ordered the COC were invited to a research visit within 18 weeks of ordering. At the visit, height and weight were measured. BP was measured with the participant sitting down having rested for ten minutes, with the arm supported at heart level. Two cuff sizes were available, dependent on participant's arm circumference. Participants were asked to refrain from talking and crossing their legs during the measurements. Three readings were taken at 10-minute intervals using a clinically validated, automated sphygmomanometer (Omron M6) [12]. During this research visit, participants completed two surveys, documenting demographic information and strategies used to obtain their self-reported measurements.

2.3 Data analysis

To compare self-reported with researcher-measured values, we report Kappa coefficients, as well as sensitivity, specificity, positive predictive value (PPV) and negative predictive value (NPV), of BP and BMI. The mean of the BP readings was used as the researcher-measured reading. Self-reported and researcher-measured BPs were classified as either UKMEC 1&2 or UKMEC 3&4. We have chosen to report sensitivity, specificity, PPV and NPV of both BMI and BP, although we recognize that since data collection was completed, guidance has changed such that BP readings as measured by our researchers are no longer the 'gold standard' for diagnosing hypertension [13]. Limitations of using researcher measurements as the 'true' BP value are considered the discussion.

The primary outcome, for which the study was powered, was the kappa coefficient for self-reported vs researcher-measured BMI and BP. Three hundred and sixty-five participants were needed to achieve 90% power to detect a true kappa value in a test of a null hypothesis (H0): Kappa = 0.7 vs an alternative hypothesis (H1): Kappa >/= 0.9. This was based on a significance level of 0.05 [14].

We used paired t-tests to calculate mean differences between self-reported and researcher-measured weight, height and BMI, systolic and diastolic BP readings. We report Pearson's correlation coefficients for the level of correlation and present Bland-Altman plots with mean differences and 95% limits of agreement, defined as ± 1.96 x (standard deviation of the difference). Analyses were completed using STATA (Version 15) and Bland Altman plots were created using SPSS (Version 24).

Participants were asked to describe how they obtained height, weight and BP measurements for their order. We classified these processes as clinically appropriate or inappropriate. Clinically appropriate was defined as a reading taken with a BP measurement device, or taken at a clinical location or pharmacy, or a manual reading taken by someone with the appropriate technical skill, within 12 months prior to ordering.

For peer review only - http://bmjopen.bmj.com/site/about/guidelines.xhtml

2.4 Public and patient involvement (PPI)

Patients and the public participated in the identification of the importance of the research question for this study and the nested qualitative study [15]. Nationally significant PPI, endorsed by the UK Faculty of Sexual and Reproductive Health Care, found that among the general population 'research to evaluate which interventions increase uptake and continuation rates of effective contraceptive methods' was the top priority for UK academic contraceptive research [16]. Reflecting this, ahead of developing the study protocol, the research team conducted their own PPI prioritization exercise with two focus groups of contraceptive service users, which identified research into online service delivery as a priority. The logic model for the intervention was similarly produced with involvement from patients and the public including interviews with 21 stakeholders [5]. Intervention development involved over 100 sexual health service users through a process of human-centred design.

3 Results

3.1 Participants

Between August 2017 and August 2019, 1228 individuals ordered COC online, of which 835 were eligible for the study and 368 (44.1%) attended a research visit (figure 1). Median time between COC order and visit was 20 days (IQR: 14-30).

For peer review only - http://bmjopen.bmj.com/site/about/guidelines.xhtml

Table 1: Sociodemographic characteristics, contraceptive and online service use of population and sample

	Eligible population n = 835, %[a]	Non-participants n = 470, %	Study participants n = 365, %	
**Sociodemographic characteristics**				
**Age (years)** [a] **mean**	24.9	24.9	24.8	0.55[d]
18-19	86 (10.3)	57 (12.1)	29 (7.9)	0.05 [d]
20-24	360 (43.1)	185 (39.3)	175 (48.0)	
25-34	355 (42.5)	208 (44.3)	147 (40.3)	
35+	34 (4.1)	20 (4.3)	14 (8.8)	
**Ethnicity** [a]				
White English/ Welsh/ Scottish/ Northern Irish/British/ Other	524 (62.7)	303 (64.5)	221 (60.6)	0.10 [d]
Black African/ Caribbean/ British/ Other	113 (13.5)	56 (11.9)	57 (15.6)	
Asian/ Asian British	86 (10.3)	53 (11.3)	33 (9.0)	
Mixed/ multiple ethnic groups	76 (9.1)	34 (7.2)	42 (11.5)	
Other ethnic groups	31 (3.7)	20 (4.3)	10 (2.7)	
Not known/ prefer not to say	6 (0.72)	4 (0.85)	2 (0.55)	
**Index of multiple deprivation quintile** [ab]				
1 (most deprived)	282 (34.2)	151 (32.7)	131 (36.2)	0.44 [d]
2	335 (40.7)	187 (40.5)	148 (40.9)	
3	155 (18.8)	89 (19.2)	66 (18.2)	
4	42 (5.1)	29 (6.3)	13 (3.6)	
5 (least deprived)	10 (1.2)	6 (1.3)	4 (1.1)	
**Qualifications (attained or studying towards)** [c]				
No academic qualifications			1 (0.27)	
GCSES (or equivalent level)			13 (3.6)	
AS/A Levels (or equivalent level)			34 (9.3)	
Higher education qualifications (or equivalent level)			316 (86.5)	
Not sure or other			1 (0.27)	
**Employment status** [c]				
Employed			265 (72.6)	
Parent/carer			2 (0.6)	
Student			93 (25.5)	
Unemployed			5 (1.3)	
**Contraceptive history and use**				
**Used oral contraception before?** [c]				
Yes			352 (96.4)	
No			13 (3.6)	
**Taken combined oral contraceptive (COC) before?** [c]				
Yes	779 (93.3)	428 (91.1)	351 (96.2)	0.003 [d]
No	56 (6.7)	42 (8.9)	14 (3.8)	
**Reason for taking the pill?** [c]				
To prevent pregnancy			302 (82.7)	
For other reasons			40 (11.0)	
To both prevent pregnancy and for other reasons			23 (6.3)	
**Online service use**				
**Ordered COC from online service before?** [a]				
Yes			94 (25.7)	
No			271 (74.3)	
**Prescribed COC from online service?**				
Yes	726 (86.9)	405 (86.2)	321 (87.9)	0.45 [d]
No	109 (13.1)	65 (13.8)	44 (12.1)	

[a] Reported by user at time of order with the online service
[b] Index of multiple deprivation data unavailable for 11 users
[c] Self-reported by participant at research visit, otherwise not collected by the online provider
[d] To compare demographic information, pill and service use between eligible users who did and did not participate in the study, t- and chi-squared tests were carried out. P values are given to 1 dp

For peer review only

Participants had a mean age of 24 years, the majority (221/365, 60.6%) were White British and 86.6% (316/365) had higher educational qualifications. Almost all (351/365, 96.2%) had used COC before, 74.3% (271/365) had not ordered COC online before, and 87.9% (321/ 365) were prescribed the COC after a full assessment of medical eligibility. Eligible users who participated in the study were similar to those who did not although the proportion of new users was higher among non-participants (table 1).

3.2 Strategies used to obtain measurements

The majority of participants (93.4%, 341/365) reported feeling confident self-reporting height, weight and BP, while 6.6% (24/365) did not. Of 365 participants, 327 provided data on their process of BP measurement that made classification of clinical appropriateness possible, and 90.5% of these (296/327) reported processes that were clinically appropriate (supplementary material 1).

Half of participants (50.1%, 181/361) measured their weight at home, 31.0% (112/361) reported a measurement from a clinic, 3.3% (12/361) from a pharmacy, 14.7% (53/361) from non-clinical setting, such workplace, gym or university, and 0.8% (3/361) participants reported estimating their weight measurements.

3.3 Agreement between BP and BMI readings

Kappa statistics suggest that the agreement between self-reported and researcher-measured BMI data was substantial (0.72 [95% confidence intervals [CI] 0.42, 1.00]), but agreement between self-reported and researcher-measured BP was poor (0.06 [95% CI -0.11, 0.23]). Two-by-two tables from which agreement was derived are presented in supplementary material B and C, full results are presented in table 2. Self-reported height and weight readings identified 80.0% (95% CI 28.4 - 99.5) of individuals with a researcher-measured BMI corresponding to UKMEC 3 or 4. Self-reported BP readings identified 9.1% (95% CI 28.4 - 99.5) of those with a researcher-measured BP corresponding to UKMEC 3 or 4. The specificity of self-reported BMI and BP measurements was high at 99.4% (95% CI 98.0- 99.9), and 97.2% (95% CI 94.9 - 98.6) respectively. Of the ten participants who were found to have self-reported BP UKMEC 1 or 2, but measured as a 3 or 4, eight reported clinically appropriate BP measurement processes and two reported unclassifiable processes.

For peer review only

For peer review only - http://bmjopen.bmj.com/site/about/guidelines.xhtml

Table 2: Agreement between self-reported and researcher-measured values

	N	Sensitivity % (95% CI)	Specificity % (95% CI)	Positive predictive value % (95% CI)	Negative predictive value % (95% CI)	Cohen's Kappa coefficient	
						Kappa coefficient (95% CI) *	P value
**UKMEC categorisation for BMI (1&2 vs 3&4)**	365	80.0 (28.4, 99.5) %	99.4 (98.0, 99.9) %	66.7 (22.3, 95.7) %	99.7 (98.5, 100.00) %	0.72 (0.42, 1.00)	0.0000
**Blood pressure (<140/90 vs > or= 140/90)**	365	9.1 (0.23, 41.3) %	97.2 (94.9, 98.6) %	9.1 (0.23, 41.3) %	97.2 (94.9, 98.6) %	0.06 (-0.11, 0.23)	0.1156

Self-reported weight was on average 1.8 kg lower (95% CI -2.4, -1.2) and the resulting BMI on average 0.70 kg/cm^2 lower (95% CI -0.94, -0.47) than researcher-measured readings. Systolic BP was 8mmHg higher (95% CI 7.1, 9.6) in the self-reported data than researcher-measured data. Average differences for height were <1mm (95% CI 0.0002, 0.008) and for diastolic BP were <1mmHg (95% CI -0.54, 1.44) (see table 3).

Table 3: Mean differences between self-reported and measured values; Pearson's correlation coefficient and Bland Altman limits of agreement

	Mean (SD)		Mean difference (95% CI); SD	Pearson's correlation coefficient	Bland Altman limits of agreement
	Self-reported values	Measured values			
**Height (m)**	1.65 (0.07)	1.65 (0.06)	0.004 (0.000, 0.008); 0.038	0.844	-
**Weight (kg)**	62.7 (10.4)	64.5 (12.4)	-1.8 [a] (-2.4, -1.2); 5.8	0.883	-
**Body mass index (kg/m2)**	22.9 (3.6)	23.6 (4.1)	-0.70 [a] (-0.94, -0.47); 2.3	0.832	- 5.2, 3.8
**Systolic blood pressure (mmHg)**	115.8 (10.4)	107.5 (9.7)	8.36 [a] (7.1, 9.6); 11.9	0.300	-15.4 to 32.1
**Diastolic blood pressure (mmHg)**	74.8 (8.5)	74.3 (7.4)	0.45 (-0.54, 1.4); 9.6	0.276	-18.8, 19.7

[a] *P* < 0.05, paired t test, difference between self-reported and measured values versus Ho: mean(diff) =0

Bland Altman plots (figures 2-4) for BMI and systolic and diastolic BP illustrate the degree of agreement between self-reported and researched-measured values [17]. Normal distribution of the differences was verified with a histogram. Limits of agreement are wider in systolic and diastolic BP than in BMI (also see table 3). The plots show that for both BP and BMI there was no trend in differences between measurements; participants with both a high and low mean BP or BMI from self-reported and researcher measurements, were found to have variable differences in self-reported and measured BP or BMI.

4 Discussion

4.1 Key findings

For peer review only - http://bmjopen.bmj.com/site/about/guidelines.xhtml

For peer review only

Self-reported BMI was found to have substantial agreement with researcher-measured BMI, unlike self-reported BP, which had poor agreement. The majority of participants undertook a clinically acceptable process in obtaining self-reported BP. On average, participants slightly under-reported their weight and over-reported their systolic BP. Bland Altman plots showed better agreement for BMI than for systolic and diastolic BP. The plots also showed no trends or systemic variation in differences between self-reported and researcher measurements.

4.2 Where this sits within the literature

Research from Australia, Finland and Sweden has found similar, substantial agreement for self-reported and researcher/clinician-measured weight and height for populations of all ages [18–20]. Studies comparing self-reported and researcher/clinician-measured BP have found varying levels of agreement, with higher agreement often found older populations [21–23]. A large cohort study (n=1537) in New Zealand found kappa between self-reported and measured BP in 18-44 year olds to be 0.26 (p <0.001), with a sensitivity of 18.1% and a specificity of 99.1% [21]. Within the same study, those aged 45-64 had an agreement of 0.49 (p <0.001), a sensitivity of 47.5%. A smaller study (n=200) of Italian women in their 50s found a kappa of 0.319 for self-reported compared with measured hypertension [23]. Self-measurement of BP is more accurate than clinician-measured BP for those who are hypertensive, at risk of hypertension or are pregnant, possibly because they receive support and training for correct measurement [24–26].

Findings from a nested qualitative sample within this study reflect a difference in self-reporting BMI and BP experiences, where height and weight measurements were accessible and familiar but BP readings were not. The qualitative study showed that participants worked hard to understand and measure BP through a combination of recent/past measurements, borrowed machines, health service visits and online research. This analysis suggests the importance of the acknowledgement of work required to measure BP, evidence of credible human support and a digital interface that communicates the health benefits of accurate measurement [15].

4.3 Strengths and limitations

This is the first study of its kind to observe self-reporting of BP and BMI to obtain the COC in a young, healthy population of reproductive age. It is timely as innovative, self-care interventions for SRH become more prevalent [27, 28].

We have compared self-reported and researcher-measured BP. Although, prior to contraception provision, clinicians taking single measures of blood pressure is standard, the assumption that the measurement from the research visit reflects the true BP is problematic. BP is inherently variable,

For peer review only - http://bmjopen.bmj.com/site/about/guidelines.xhtml

influenced by numerous extrinsic and intrinsic factors [29]. A systematic review of the sources of inaccuracy in the measurement of adult resting BP, identified significant directional effects from 27 sources, resulting in a range of -23.6 to +33.0 mmHg for systolic BP and -14.0 to +23.0 mmHg for diastolic BP [30]. Whilst researchers employed recommended strategies to mitigate inaccuracies in BP measurement, some factors remain irremediable, for example 'white coat syndrome', whereby researcher-, or clinician-, measured BP are artificially raised due to anxiety, or natural BP variation [31, 32]. Recently updated UK guidance recommends ambulatory or home BP monitoring involving multiple repeated measures over a day for ambulatory or at least 4 days for home BP monitoring to diagnose hypertension [13].

There was potential for self-selection bias during recruitment where users who may have estimated their BP during their order might have been unwilling to participate. To mitigate this, invitation communication, whilst providing sufficient information on the study procedure, did not emphasise validation of measurements (see supplementary material D). The study had good uptake, with 44.1% of eligible users attending a research visit, where researchers reiterated their independence from the online service.

The medium time between COC order and research visit was 20 days. This may have impacted on participants' recall of the details of recent BP measurements. This is evident in the 38 unclassifiable participant processes: 31 participants gave the date of their most recent BP measurement between their order and visit, seven were unclassifiable due to limited information in their response.

There are methodological limitations that may explain the low kappa coefficient. The kappa produced, in particular for BP, should be interpreted considering the method's paradoxes and problems. The two-by-two table for BP (supplementary material C) produced, with it's perfectly symmetrically unbalanced marginal totals, satisfies both paradoxes outlined by Feinstein and Cicchetti, which perhaps provides an additional factor explaining the low kappa in light of high percentage agreement [33].

4.4 Meaning and mechanism

Explanations for our findings include the natural variability of BP, white coat syndrome, or inaccurate self-measurement or reporting [31, 32]. Although our quantitative data does not allow us to explore whether our findings are due to the natural variability of BP or to inaccurate self-measurement or reporting, our qualitative research provides some insights [15]. Participants shared both experiences of white coat syndrome at previous consultations, and, in relation to their online COC order, difficulties obtaining a new BP measurement, remembering values from a recent reading, and

For peer review only - http://bmjopen.bmj.com/site/about/guidelines.xhtml

For peer review only

difficulties interpreting or uploading readings, as well as a single participant who recounted misreporting despite a known high BP.

Whilst BP limits of agreement are comparable to biologically plausible ranges established when estimating the impact of sources of inaccuracy [30], the clinically relevant factor for remote COC prescription is not the magnitude of these limits, but whether they shift BP from a non-contraindication to a contraindication. Nevertheless, at their widest, such limits would trigger clinical concern and might initiate a conversation about process and accuracy of measurement.

The difference in findings for BP and BMI might reflect differences in familiarity with their measurement. Fifty years ago, accurate equipment for measuring weight were rare in households and most people did not know their current weight. BP monitors are now transitioning from a technology predominantly available in health care settings to one that is increasingly available at home. Strategies to increase accuracy of reporting could focus on improving understanding of BP and access to measurement.

5 Conclusions and recommendations

Online contraceptive services provide a convenient alternative for oral contraception users which may improve uptake and continuation [5]. Accuracy of self-reported height, weight and BP is important to ensure appropriate, safe COC prescription online, since BMI and BP may contraindicate use [6, 7].

Whilst self-reported BMI was found to have substantial agreement with researcher-measured BMI, self-reported BP was shown to have poor agreement with researcher-measured BP. These differences may be due to the natural variability of BP, white coat syndrome, methodological limitations associated with kappa or by inaccurate self-measurement and reporting.

As online contraceptive services are increasingly prevalent and self-measured BP has been shown to be valid in other populations, strategies to improve BP self-measurement for remote prescription of COC are needed. In response to this study, in collaboration with the study team, SH:24 have completed a process of user-centred design that aims to create a 'safe and supportive enabling environment' to maximise the benefits and minimise the risks of self-care, acknowledging the responsibility of service providers in this respect [27, 34]. The design process suggested innovations that include: building skills for self-monitoring in face-to-face care when BP is measured by clinicians; providing home BP monitors with support to use them; improved online information on the importance of BP measurements and consequences of inaccurate reporting; credible clinical support as required; issuing a short supply for the first order with information on how to obtain a BP

For peer review only - http://bmjopen.bmj.com/site/about/guidelines.xhtml

measurement [15, 35]. These strategies may improve the accuracy of BP reporting and we recommend further research to evaluate them.

Acknowledgements

We thank all users of the online service who took part in this study. We thank SH:24 for collaborating on the project, in particular the clinical team, designers and data analyst at the online service for their support with recruitment and data collection.

Ethical considerations

The East Midlands – Leicester Research Ethics Committee granted ethical approval to the project (Evaluation of online provision of oral contraceptives to measure: accuracy of self-reported height, weight and blood pressure; essential information transfer and user experience, reference 17/EM/0181) on 6th June 2017. Before participating in a research visit, users were presented with a participant information sheet, were given the opportunity to ask questions, which they had answered to their satisfaction, and then provided informed consent. Participation was voluntary, and participants were assured that the information they provided would be anonymised and kept confidential. All participants whose biometric data suggested a UKMEC category 3 or 4 or who reported contraindications to the COC during the study or indicated a need for further clinical support were referred to a local sexual health service.

Funding

This report is independent research funded by the National Institute for Health Research (Research for Patient Benefit Programme, PB-PG-0815-20009). The views expressed in this publication are those of the author(s) and not necessarily those of the NIHR or the Department for Health and Social Care. The sponsor, King's College Hospital, was not involved in study design, data collection, analysis, or interpretation of data.

Data statement

The anonymised dataset is available from the authors on request.

Competing interests

Since completion of this study, HM has worked at the online service on a different research project. PB is clinical director of the online service studied and is one of the prescribers within this service. AM has also been employed by the online service as a bank clinical support midwife. This work is part of a process of research on innovation at SH:24 where all innovation is the subject of research to ensure that learning is shared widely. SH:24 is a 'not for profit' organisation that provides health

For peer review only - http://bmjopen.bmj.com/site/about/guidelines.xhtml

For peer review only

services to the National Health Service and sharing learning through innovation and research is one of the principles of the organisation.

Author contributions

Hannah McCulloch contributed to data collection, led the data analysis, and drafted the manuscript.

Alessandra Morelli contributed to data collection and data analysis and commented on the manuscript.

Caroline Free contributed to the conception and design of the study, contributed to data analysis and commented on the manuscript.

Jonathan Syred contributed to the conception and design of the study, contributed to data collection and commented on the manuscript.

Riley Botelle contributed to data collection and commented on the manuscript.

Paula Baraitser contributed to the conception and design of the study, oversaw data collection, contributed to data analysis and commented on the manuscript.

Figure headings

Figure 1: Recruitment Flow Chart for users of the online service

Figure 2. Bland Altman plot of the differences between self-reported and researcher-measured BMI compared with the mean self-reported and researcher-measured BMI. The central line represents the mean difference between self-reported and researcher-measured BMI. The upper and lower boundaries represent the 95% limits of agreement.

Figure 3: Bland Altman plot of the differences between self-reported and researcher-measured systolic BP compared with the mean self-reported and researcher-measured systolic BP. The central line represents the mean difference between self-reported and researcher-measured systolic BP. The upper and lower boundaries represent the 95% limits of agreement.

Figure 4. Bland Altman plot of the differences between self-reported and researcher-measured diastolic BP compared with the mean self-reported and researcher-measured diastolic BP. The central line represents the mean difference between self-reported and researcher-measured diastolic BP. The upper and lower boundaries represent the 95% limits of agreement.

References

[1] French RS, Gibson L, Geary R, et al. Changes in the prevalence and profile of users of contraception in Britain 2000–2010: evidence from two National Surveys of Sexual Attitudes

For peer review only

and Lifestyles. *BMJ Sex Reprod Heal* 2020; bmjsrh-2019-200474.

[2] Royal College of Obstetricians and Gynaecologists. *Better for Women: Improving the health and wellbeing of girls and women*, https://www.rcog.org.uk/en/news/campaigns-and-opinions/better-for-women/ (2019).

[3] Wilson E, Free C, Morris TP, et al. Internet-accessed sexually transmitted infection (e-STI) testing and results service: A randomised, single-blind, controlled trial. *PLOS Med* 2017; 14: e1002479.

[4] Wilson E, Baraitser P, Free C. Does internet-accessed STI (e-STI) testing increase testing uptake for chlamydia and other STIs among a young population who have never tested? Secondary analyses of data from a randomised controlled trial. *Sex Transm Infect* 2019; 0: 1–6.

[5] Rezel-Potts E, Free C, Syred J, et al. Expanding choice through online contraception: A theory of change to inform service development and evaluation. *BMJ Sex Reprod Heal*. Epub ahead of print 2019. DOI: 10.1136/bmjsrh-2018-200297.

[6] Faculty of Sexual and Reproductive Healthcare. *UK Medical eligibility criteria for contraceptive use: UKMEC 2016 (Updated 2019)*.

[7] World Health Organisation. *Medical Eligibility Criteria for Contraceptive Use*, https://www.who.int/reproductivehealth/publications/family_planning/Ex-Summ-MEC-5/en/ (2015).

[8] SH:24. Programme overview, https://sh24.org.uk/.

[9] Public Health England. Sexual and Reproductive Health Profiles, https://fingertips.phe.org.uk/profile/sexualhealth/data#page/0/gid/8000057/pat/6/par/E12000007/ati/102/are/E09000028 (2020, accessed 4 March 2020).

[10] Lambeth Southwark and Lewisham Public Health Departments. *Lambeth, Southwark and Lewisham Sexual and Reproductive Health Strategy 2019–24*. London, https://www.lambeth.gov.uk/sites/default/files/yh-lsl-sexual-health-strategy_0.pdf (2019).

[11] General Medical Council. *Good practice in prescribing and managing medicines and devices*, https://www.gmc-uk.org/Prescribing_guidance.pdf_59055247.pdf (2013).

[12] Topouchian J, Agnoletti D, Blacher J, et al. Validation of four devices: Omron M6 Comfort, Omron HEM-7420, Withings BP-800, and Polygreen KP-7670 for home blood pressure measurement according to the European society of hypertension international protocol. *Vasc Health Risk Manag* 2014; 10: 33–44.

[13] National Institute for Health and Care Excellence. *Hypertension in adults: diagnosis and management*, https://www.nice.org.uk/guidance/ng136.

[14] Sim J, Wright CC. The Kappa Statistic in Reliability Studies: Use, Interpretation, and Sample Size Requirements. *Phys Ther* 2005; 85: 257–268.

[15] Baraitser P, McCulloch H, Morelli A, et al. How do users of a digital-only contraceptive service provide biometric measurements and what does this teach us about safe and effective online care? A qualitative interview study. *BMJ Open*; 10. Epub ahead of print 2020. DOI: 10.1136/bmjopen-2020-037851.

[16] Faculty of Sexual & Reproductive Healthcare (FSRH). *Collaboration, Choice, Care: The Contraception Priority Setting Partnership (PSP)*, https://www.fsrh.org/documents/fsrh-contraception-psp-report-2018-jla/ (2018, accessed 29 June 2021).

For peer review only - http://bmjopen.bmj.com/site/about/guidelines.xhtml

[17] Giavarina D. Understanding Bland Altman analysis. *Biochem Medica* 2015; 25: 141–151.

[18] Davies A, Wellard-Cole L, Rangan A, et al. Validity of self-reported weight and height for BMI classification: A cross-sectional study among young adults. *Nutrition* 2020; 71: 110622.

[19] Dahl A, Hassing L, Fransson E, et al. Agreement between self-reported and measured height, weight and body mass index in old age—a longitudinal study with 20 years of follow-up. *Age Ageing* 2010; 39: 445–451.

[20] Tuomela J, Kaprio J, Sipilä PN, et al. Accuracy of self-reported anthropometric measures — Findings from the Finnish Twin Study. *Obes Res Clin Pract* 2019; 13: 522–528.

[21] Taylor A, Grande ED, Gill T, et al. Comparing self-reported and measured high blood pressure and high cholesterol status using data from a large representative cohort study. *Aust N Z J Public Health* 2010; 34: 394–400.

[22] Ning M, Zhang Q, Yang M. Comparison of self-reported and biomedical data on hypertension and diabetes: Findings from the China Health and Retirement Longitudinal Study (CHARLS). *BMJ Open*; 6. Epub ahead of print 2016. DOI: 10.1136/bmjopen-2015-009836.

[23] Villarini M, Acito M, Gianfredi V, et al. Validation of Self-Reported Anthropometric Measures and Body Mass Index in a Subcohort of the DianaWeb Population Study. *Clin Breast Cancer* 2019; 19: e511–e518.

[24] McManus RJ, Wood S, Bray EP, et al. Self-monitoring in hypertension: A web-based survey of primary care physicians. *J Hum Hypertens* 2014; 28: 123–127.

[25] Hinton L, Tucker KL, Greenfield SM, et al. Blood pressure self-monitoring in pregnancy (BuMP) feasibility study; A qualitative analysis of women's experiences of self-monitoring. *BMC Pregnancy Childbirth*; 17. Epub ahead of print 19 December 2017. DOI: 10.1186/s12884-017-1592-1.

[26] Tucker KL, Taylor KS, Crawford C, et al. Blood pressure self-monitoring in pregnancy: Examining feasibility in a prospective cohort study. *BMC Pregnancy Childbirth*; 17. Epub ahead of print 28 December 2017. DOI: 10.1186/s12884-017-1605-0.

[27] Narasimhan M, Allotey P, Hardon A. Self care interventions to advance health and wellbeing: A conceptual framework to inform normative guidance. *BMJ*; 365. Epub ahead of print 2019. DOI: 10.1136/bmj.l688.

[28] World Health Organization (WHO). WHO Consolidated Guideline on Self-Care Interventions for Health: Sexual and Reproductive Health and Rights, https://apps.who.int/iris/bitstream/handle/10665/325480/9789241550550-eng.pdf?ua=1 (2020).

[29] Pickering TG. Blood pressure variability and ambulatory monitoring. *Current opinion in nephrology and hypertension* 1993; 2: 380–385.

[30] Kallioinen N, Hill A, Horswill MS, et al. Sources of inaccuracy in the measurement of adult patients' resting blood pressure in clinical settings: A systematic review. *Journal of Hypertension* 2017; 35: 421–441.

[31] McGrath BP. Ambulatory blood pressure monitoring and 'white coat' hypertension: Saving costs. *Medical Journal of Australia* 2002; 176: 571–572.

[32] Turner MJ, Van Schalkwyk JM. Blood pressure variability causes spurious identification of hypertension in clinical studies: A computer simulation study. *Am J Hypertens* 2008; 21: 85–91.

For peer review only

For peer review only - http://bmjopen.bmj.com/site/about/guidelines.xhtml

[33] Feinstein AR, Cicchetti D V. High agreement but low Kappa: I. the problems of two paradoxes. *J Clin Epidemiol* 1990; 43: 543–549.

[34] Ferguson L, Fried S, Matsaseng T, et al. Human rights and legal dimensions of self care interventions for sexual and reproductive health. *BMJ*; 365. Epub ahead of print 2019. DOI: 10.1136/bmj.l1941.

[35] Baraitser P, McCulloch H, Shather Z, et al. Supporting blood pressure measurement prior to online prescription of the combined oral contraceptive. *BMJ Sex Reprod Heal*. Epub ahead of print 2021. DOI: 10.1136/bmjsrh-2020-200887.

For peer review only

For peer review only - http://bmjopen.bmj.com/site/about/guidelines.xhtml

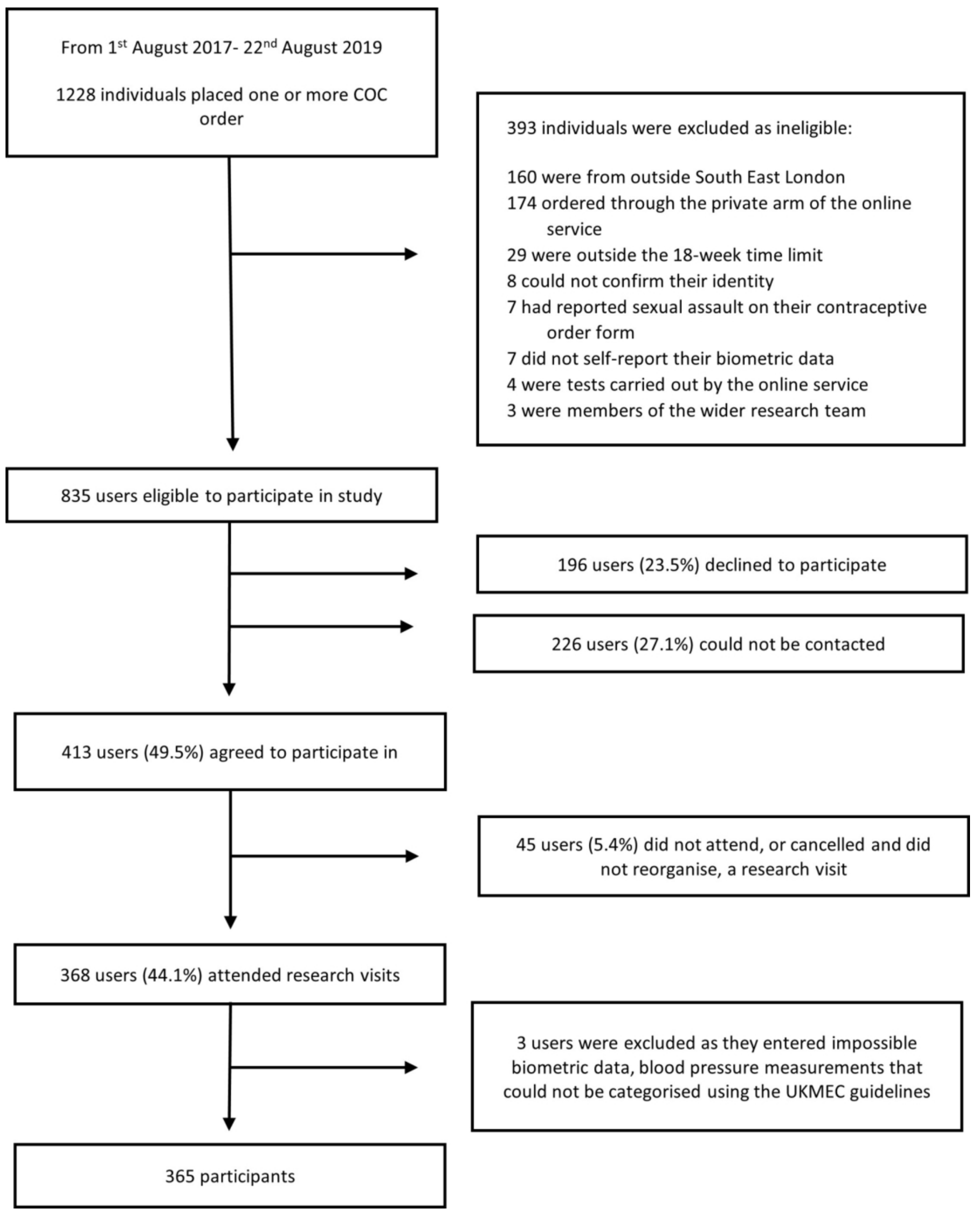

Figure 1: Recruitment Flow Chart for users of the online service

154x191mm (300 x 300 DPI)

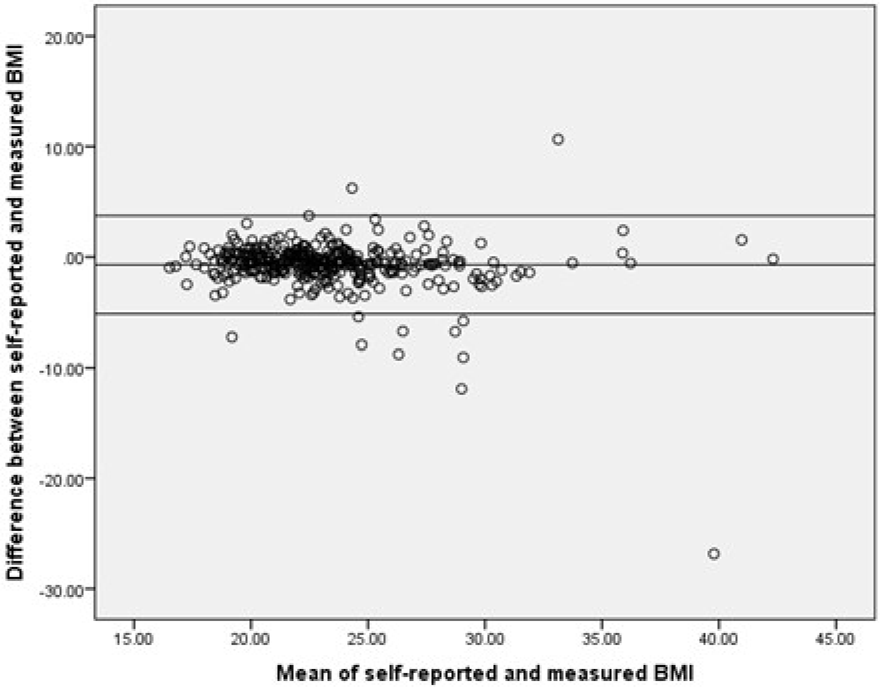

Figure 2. Bland Altman plot of the differences between self-reported and researcher-measured BMI compared with the mean self-reported and researcher-measured BMI. The central line represents the mean difference between self-reported and researcher-measured BMI. The upper and lower boundaries represent the 95% limits of agreement.

119x93mm (96 x 96 DPI)

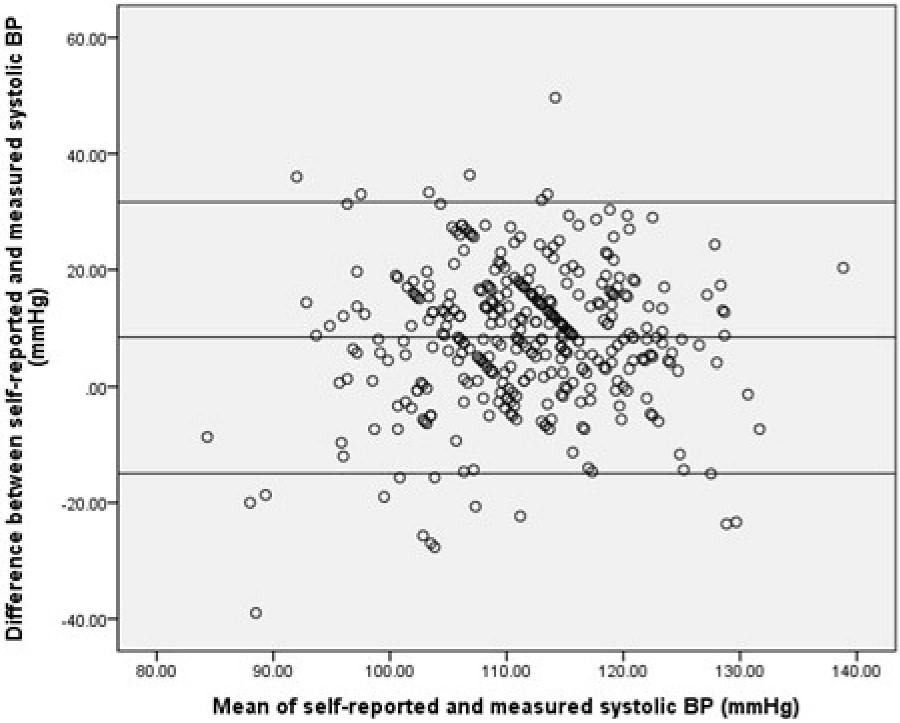

Figure 3: Bland Altman plot of the differences between self-reported and researcher-measured systolic BP compared with the mean self-reported and researcher-measured systolic BP. The central line represents the mean difference between self-reported and researcher-measured systolic BP. The upper and lower boundaries represent the 95% limits of agreement

122x97mm (96 x 96 DPI)

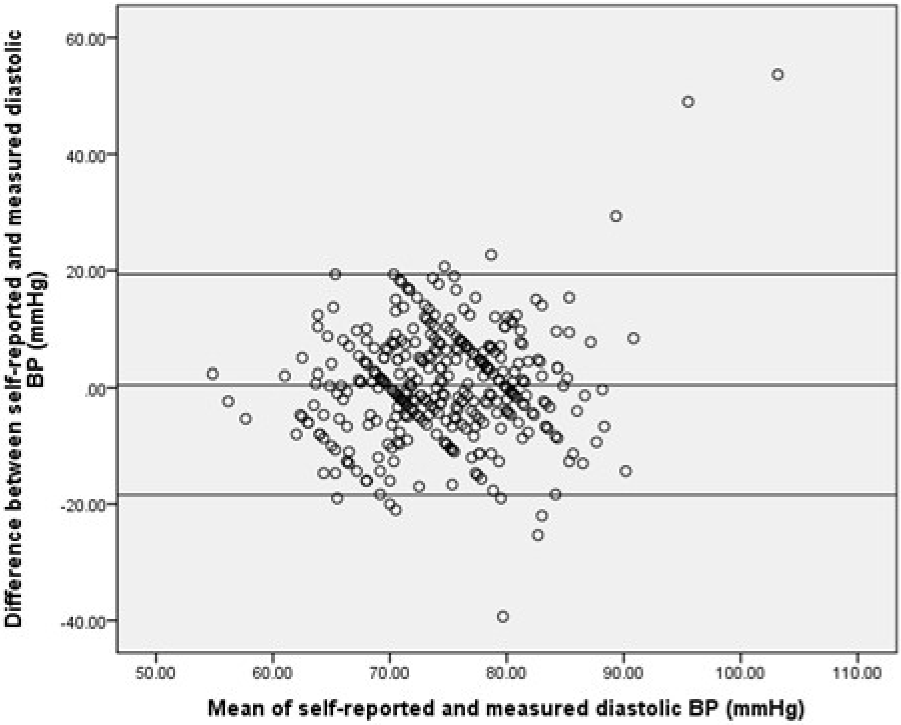

Figure 4. Bland Altman plot of the differences between self-reported and researcher-measured diastolic BP compared with the mean self-reported and researcher-measured diastolic BP. The central line represents the mean difference between self-reported and researcher-measured diastolic BP. The upper and lower boundaries represent the 95% limits of agreement.

122x98mm (96 x 96 DPI)

Supplementary material

A. Process of BP measurement and examples from free text answers

Process of blood pressure measurement	Example
Unclassifiable	- I have a blood pressure monitor at home (date given between order and research visit)<br>- "My blood pressure was measured by a colleague at work (12 days before order)<br>- "[South East London Clinic] (date given between order and research visit)"<br>- "I have a nurse at my office that offer blood pressure testing so I used the facilities provided to check (date given between order and research visit)"<br>- "I visited the pharmacy stand in boots. They took me to a private room and took my blood pressure for me (date given between order and research visit)"
Clinically appropriate	- "From my GP when I was first prescribed the pill (3 months before order)"<br>- "The nurse in the clinic took my blood pressure (same day as order)"<br>- "Inherited blood pressure machine from relative with long term illness who no longer needed it. Used this at home to obtain measure (4 days before order)<br>- "I visited my aunts house to get my blood pressure reading since she has a machine at home (day before order)<br>- "I went to my local pharmacy where they had a self checker machine that calculated my blood pressure, weight and height (same day as order)"
Clinically inappropriate	- "At the GP before getting a repeat prescription of the pill, which was measured normally (18 months before order)"<br>- "A bupa nurse came into my place of work and offered a health check. My blood pressure was taken there (2 years before order)<br>- "I looked up what a healthy blood pressure would be on NHS website and put in that blood pressure when on the sh:24 website"<br>- "Estimated. Had a blood pressure machine but feel I did not use it correctly"<br>- "Googled healthy blood pressure"

For peer review only - http://bmjopen.bmj.com/site/about/guidelines.xhtml

B. Self-reported and researcher-measured BMI

		Measured BMI		
		**UKMEC 1&2 <35.0 kg/m**	**UKMEC 3&4 > or= 35 kg/m**	**Total**
**Self-reported BMI**	**UKMEC 1&2 <35.0 kg/m**	358	1	359
	**UKMEC 3&4 > or= 35 kg/m**	2	4	6
	**Total**	360	5	365

C. Self-reported and researcher-measured BP

		Researcher-measured blood pressure (mmHg)		
		**< 140/90**	**> or = 140/90**	**Total**
**Self-reported blood pressure (mmHg)**	**<140/90**	344	10	354
	**> or= 140/90**	10	1	11
	**Total**	354	11	365

D. SMS invitation message

“Hello. SH:24 are working with King's College Hospital to improve contraception services. King's would like to invite you to take part in their research - they will thank you for your time with £45. The research would involve meeting a member of the King's team to allow them to check your height, weight and blood pressure. You will also be asked to complete a short questionnaire (this should take no more than 40 mins in total). Text back NO if you don't want to receive a call back from the King's research team. Thanks, SH:24"

For peer review only

Agreement between self-reported and researcher-measured height, weight and blood pressure measurements for online prescription of the combined oral contraceptive pill: an observational study

Journal:	*BMJ Open*
Manuscript ID	bmjopen-2021-054981.R1
Article Type:	Original research
Date Submitted by the Author:	24-Jan-2022

Complete List of Authors:	McCulloch, Hannah; King's College London Faculty of Life Sciences and Medicine, King's Centre for Global Health and Health Partnerships<br>Morelli, Alessandra; King's College London Faculty of Life Sciences and Medicine, King's Centre for Global Health and Health Partnerships<br>Free, Caroline; London School of Hygiene and Tropical Medicine Faculty of Epidemiology and Population Health, Department of Population Health<br>Syred, Jonathan; King's College London Faculty of Life Sciences and Medicine, King's Centre for Global Health and Health Partnerships<br>Botelle, Riley; King's College London Faculty of Life Sciences and Medicine, Kings Centre for Global Health and Health Partnerships<br>Baraitser, Paula; King's College Hospital, Sexual Health; SH:24 CIC

**Primary Subject Heading**:	Reproductive medicine
Secondary Subject Heading:	Public health, Sexual health
Keywords:	SEXUAL MEDICINE, REPRODUCTIVE MEDICINE, PUBLIC HEALTH

SCHOLARONE™
Manuscripts

I, the Submitting Author has the right to grant and does grant on behalf of all authors of the Work (as defined in the below author licence), an exclusive licence and/or a non-exclusive licence for contributions from authors who are: i) UK Crown employees; ii) where BMJ has agreed a CC-BY licence shall apply, and/or iii) in accordance with the terms applicable for US Federal Government officers or employees acting as part of their official duties; on a worldwide, perpetual, irrevocable, royalty-free basis to BMJ Publishing Group Ltd ("BMJ") its licensees and where the relevant Journal is co-owned by BMJ to the co-owners of the Journal, to publish the Work in this journal and any other BMJ products and to exploit all rights, as set out in our licence.

The Submitting Author accepts and understands that any supply made under these terms is made by BMJ to the Submitting Author unless you are acting as an employee on behalf of your employer or a postgraduate student of an affiliated institution which is paying any applicable article publishing charge ("APC") for Open Access articles. Where the Submitting Author wishes to make the Work available on an Open Access basis (and intends to pay the relevant APC), the terms of reuse of such Open Access shall be governed by a Creative Commons licence – details of these licences and which Creative Commons licence will apply to this Work are set out in our licence referred to above.

Other than as permitted in any relevant BMJ Author's Self Archiving Policies, I confirm this Work has not been accepted for publication elsewhere, is not being considered for publication elsewhere and does not duplicate material already published. I confirm all authors consent to publication of this Work and authorise the granting of this licence.

For peer review only

Agreement between self-reported and researcher-measured height, weight and blood pressure measurements for online prescription of the combined oral contraceptive pill: an observational study

Hannah McCulloch[1], Alessandra Morelli[1], Caroline Free[2], Jonathan Syred[1], Riley Botelle[1], Paula Baraitser[134]

Author affiliations

[1] King's College London, King's Centre for Global Health and Health Partnerships, London, United Kingdom

[2] London School of Hygiene and Tropical Medicine, Department of Population Health, London, United Kingdom

[3] King's College Hospital, Camberwell Sexual Health Clinic, 94-104 Denmark Hill, SE5 9RS, London, United Kingdom

[4] SH:24, 35A Westminster Bridge Road, SE1 7JB, London, United Kingdom

Corresponding author:

Hannah McCulloch, King's College London, King's Centre for Global Health and Health Partnerships, Weston Education Centre, 10 Cutcombe Rd, SE5 9RJ London, United Kingdom; hrmcculloch@gmail.com

Title word count: 23

Abstract word count: 255

Strengths and limitations statement: 121

Full article word count: 2967

For peer review only - http://bmjopen.bmj.com/site/about/guidelines.xhtml

Abstract

Title: Agreement between self-reported and researcher-measured height, weight and blood pressure measurements for online prescription of the combined oral contraceptive pill: an observational study

Objective(s): To compare agreement between self-reported height, weight and blood pressure measurements submitted to an online contraceptive service with researcher-measured values and document strategies used for self-reporting.

Design: An observational study.

Setting: An online sexual health service which provided the combined oral contraceptive pill, free of charge, to users in Southeast London, England.

Participants: Between August 2017 and August 2019, 365 participants were recruited.

Primary and secondary outcome measures: The primary outcome, for which the study was powered, was the agreement between self-reported and researcher-measured body mass index and blood pressure measurements, compared using kappa coefficients. Secondary measures of agreement included sensitivity, specificity, and Bland Altman plots. The study also describes strategies used for self-reporting and classifies their clinical appropriateness.

Results: 327 participants fully described their process of blood pressure measurement with 296 (90.5%) classified as clinically appropriate. Agreement between self-reported and researcher-measured body mass index was substantial (0.72 [95% confidence intervals [CI] 0.42, 1.0]), but poor for blood pressure (0.06 [95% CI -0.11, 0.23]). Self-reported height and weight readings identified 80.0% (95% CI 28.4 - 99.5) of individuals with a researcher-measured high body mass index (≥ than 35kg/m2) and 9.1% (95% CI 0.23, 41.3) of participants with a researcher-measured high blood pressure (≥ 140/90 mmHg).

Conclusion: In this study, self-reported metrics were good predictors of researcher-measured body mass index but not blood pressure. This may be due to the inherent variability of blood pressure, overdiagnosis of hypertension by researchers due to 'white coat syndrome' or inaccurate self-reporting. Strategies to improve self-reporting of blood pressure for remote prescription of the combined pill are needed.

Strengths and limitations of this study

- This is the first study to observe self-reporting of blood pressure and body mass index to obtain the combined oral contraceptive pill in a healthy population of reproductive age.

For peer review only - http://bmjopen.bmj.com/site/about/guidelines.xhtml

- We compared blood pressure and body mass index measurements self-reported by users online with researcher-measured readings, taken on average 20 days later.
- This study's assumption that researcher measurements reflect true blood pressure is problematic, however, clinicians taking single measures of blood pressure is standard practice in contraceptive provision
- We recognise the potential for self-selection bias in recruitment, where users who may have estimated their blood pressure during their order might have been unwilling to participate, and for healthy user effect since the majority of participants had previous experience of taking the pill

Key words: Online sexual health services, contraception, self-care, self-reported biometric data, remote prescribing, blood pressure

For peer review only

<u>Full article</u>

1. Introduction

In the United Kingdom (UK), 29.5% of sexually active women of reproductive age use oral contraceptives (1). Whilst contraception is available free of cost in the UK, research has found that 37.0% of women have difficulty accessing contraceptive services (2).

For peer review only - http://bmjopen.bmj.com/site/about/guidelines.xhtml

Online sexual health services, offering tests for sexually transmitted infections (STIs) ordered online and posted home, with self-taken samples posted to laboratories and results communicated remotely, have become part of the UK sexual health economy. Online services increase access to testing, including amongst those who have never tested before (3,4).

Online services might facilitate access to contraception, enabling users to order oral contraception with remote prescribing and postal delivery (5). Remote prescription of the combined oral contraceptive (COC) requires a medical history and self-reported height, weight and blood pressure (BP). The accuracy of these biometric measurements is important to ensure appropriate, safe prescription.

The UK and World Health Organisation (WHO) medical eligibility criteria (UKMEC/WHOMEC) classify all contraceptive contraindications on a scale of 1-4 (6,7). A classification of 1 or 2 implies that benefits of COC use outweigh the risks, whilst a classification of 3 or 4 suggests that risks of use outweigh benefits. A body mass index (BMI) equal to or greater than 35kg/m2 or a BP consistently above 140/90 mmHg would be classified as UKMEC 3 or 4.

We studied a UK-based, online contraceptive service, SH:24, which provided COC via post after an online medical history and self-reported height, weight and BP (8). We aimed to compare self-reported BMI and BP measurements with measurements taken by researchers.

2. Materials and methods

2.1. Setting and ethical approval

SH:24 provided online sexual health services free to the residents of five areas of South East London, areas with poor sexual and reproductive health (SRH) indicators (9,10), between January 2017 and August 2019. SH:24 is an NHS-commissioned-service, registered with the UK Quality Care Commission and compliant with the General Medical Council guidance on remote prescribing (11). SH:24 was advertised through online media, and signposting from clinics. The study was given ethical approval by the East Midlands – Leicester Research Ethics Committee on 6th June 2017.

2.2. Data collection

Users who ordered the COC were invited to a research visit within 18 weeks of ordering. At the visit, height and weight were measured. BP was measured with the participant sitting down having rested for ten minutes, with the arm supported at heart level. Two cuff sizes were available, dependent on participant's arm circumference. Participants were asked to refrain from talking and crossing their legs during the measurements. Three readings were taken at 10-minute intervals using a clinically validated, automated sphygmomanometer (Omron M6) (12). During this research visit, participants

For peer review only - http://bmjopen.bmj.com/site/about/guidelines.xhtml

completed two surveys, documenting demographic information and strategies used to obtain their self-reported measurements.

2.3. Data analysis

To compare self-reported with researcher-measured values, we report Kappa coefficients, as well as sensitivity, specificity, positive predictive value (PPV) and negative predictive value (NPV), of BP and BMI. The mean of the BP readings was used as the researcher-measured reading. Self-reported and researcher-measured BPs were classified as either UKMEC 1&2 or UKMEC 3&4. We have chosen to report sensitivity, specificity, PPV and NPV of both BMI and BP, although we recognize that since data collection was completed, guidance has changed such that BP readings as measured by our researchers are no longer the 'gold standard' for diagnosing hypertension (13). Limitations of using researcher measurements as the 'true' BP value are considered the discussion.

The primary outcome, for which the study was powered, was the kappa coefficient for self-reported vs researcher-measured BMI and BP. Three hundred and sixty-five participants were needed to achieve 90% power to detect a true kappa value in a test of a null hypothesis (H0): Kappa = 0.7 vs an alternative hypothesis (H1): Kappa >/= 0.9. This was based on a significance level of 0.05 (14), and a prevalence of UKMEC 3&4 BP and BMI of 7.5%, which was estimated from contraceptive consultations at a local sexual health service in one of the study areas of South East London.

We used paired t-tests to calculate mean differences between self-reported and researcher-measured weight, height and BMI, systolic and diastolic BP readings. We report Pearson's correlation coefficients for the level of correlation and present Bland-Altman plots with mean differences and 95% limits of agreement, defined as ± 1.96 x (standard deviation of the difference). Analyses were completed using STATA (Version 15) and Bland Altman plots were created using SPSS (Version 24).

Participants were asked to describe how they obtained height, weight and BP measurements for their order. We classified these processes as clinically appropriate or inappropriate. Clinically appropriate was defined as a reading taken with a BP measurement device, or taken at a clinical location or pharmacy, or a manual reading taken by someone with the appropriate technical skill, within 12 months prior to ordering.

2.4. Public and patient involvement (PPI)

Patients and the public participated in the identification of the importance of the research question for this study and the nested qualitative study (15). Nationally significant PPI, endorsed by the UK Faculty of Sexual and Reproductive Health Care, found that among the general population 'research

For peer review only - http://bmjopen.bmj.com/site/about/guidelines.xhtml

to evaluate which interventions increase uptake and continuation rates of effective contraceptive methods' was the top priority for UK academic contraceptive research (16).Reflecting this, ahead of developing the study protocol, the research team conducted their own PPI prioritization exercise with two focus groups of contraceptive service users, which identified research into online service delivery as a priority. The logic model for the intervention was similarly produced with involvement from patients and the public including interviews with 21 stakeholders (5). Intervention development involved over 100 sexual health service users through a process of human-centred design.

3. Results

3.1. Participants

Between August 2017 and August 2019, 1228 individuals ordered COC online, of which 835 were eligible for the study and 368 (44.1%) attended a research visit (figure 1). Median time between COC order and visit was 20 days (IQR: 14-30).

For peer review only

Table 1: Sociodemographic characteristics, contraceptive and online service use of population and sample

	Eligible population n = 835, %[a]	Non-participants n = 470, %	Study participants n = 365, %	
**Sociodemographic characteristics**				
**Age (years)** [a] **mean**	24.9	24.9	24.8	0.55[d]

For peer review only - http://bmjopen.bmj.com/site/about/guidelines.xhtml

18-19	86 (10.3)	57 (12.1)	29 (7.9)	0.05 [d]
20-24	360 (43.1)	185 (39.3)	175 (48.0)	
25-34	355 (42.5)	208 (44.3)	147 (40.3)	
35+	34 (4.1)	20 (4.3)	14 (8.8)	
**Ethnicity** [a]				
White English/ Welsh/ Scottish/ Northern Irish/British/ Other	524 (62.7)	303 (64.5)	221 (60.6)	0.10 [d]
Black African/ Caribbean/ British/ Other	113 (13.5)	56 (11.9)	57 (15.6)	
Asian/ Asian British	86 (10.3)	53 (11.3)	33 (9.0)	
Mixed/ multiple ethnic groups	76 (9.1)	34 (7.2)	42 (11.5)	
Other ethnic groups	31 (3.7)	20 (4.3)	10 (2.7)	
Not known/ prefer not to say	6 (0.72)	4 (0.85)	2 (0.55)	
**Index of multiple deprivation quintile** [ab]				
1 (most deprived)	282 (34.2)	151 (32.7)	131 (36.2)	0.44 [d]
2	335 (40.7)	187 (40.5)	148 (40.9)	
3	155 (18.8)	89 (19.2)	66 (18.2)	
4	42 (5.1)	29 (6.3)	13 (3.6)	
5 (least deprived)	10 (1.2)	6 (1.3)	4 (1.1)	
**Qualifications (attained or studying towards)** [c]				
No academic qualifications			1 (0.27)	
GCSES (or equivalent level)			13 (3.6)	
AS/A Levels (or equivalent level)			34 (9.3)	
Higher education qualifications (or equivalent level)			316 (86.5)	
Not sure or other			1 (0.27)	
**Employment status** [c]				
Employed			265 (72.6)	
Parent/carer			2 (0.6)	
Student			93 (25.5)	
Unemployed			5 (1.3)	
**Contraceptive history and use**				
**Used oral contraception before?** [c]				
Yes			352 (96.4)	
No			13 (3.6)	
**Taken combined oral contraceptive (COC) before?** [c]				
Yes	779 (93.3)	428 (91.1)	351 (96.2)	0.003 [d]
No	56 (6.7)	42 (8.9)	14 (3.8)	
**Reason for taking the pill?** [c]				
To prevent pregnancy			302 (82.7)	
For other reasons			40 (11.0)	
To both prevent pregnancy and for other reasons			23 (6.3)	
**Online service use**				
**Ordered COC from online service before?** [a]				
Yes			94 (25.7)	
No			271 (74.3)	
**Prescribed COC from online service?**				
Yes	726 (86.9)	405 (86.2)	321 (87.9)	0.45 [d]
No	109 (13.1)	65 (13.8)	44 (12.1)	

[a] Reported by user at time of order with the online service
[b] Index of multiple deprivation data unavailable for 11 users
[c] Self-reported by participant at research visit, otherwise not collected by the online provider
[d] To compare demographic information, pill and service use between eligible users who did and did not participate in the study, t- and chi-squared tests were carried out. P values are given to 1 dp

Participants had a mean age of 24 years, the majority (221/365, 60.6%) were White British and 86.6% (316/365) had higher educational qualifications. Almost all (351/365, 96.2%) had used COC before, 74.3% (271/365) had not ordered COC online before, and 87.9% (321/ 365) were prescribed the COC after a full assessment of medical eligibility. Eligible users who participated in the study

For peer review only - http://bmjopen.bmj.com/site/about/guidelines.xhtml

were similar to those who did not although the proportion of new users was higher among non-participants (table 1).

3.2. Strategies used to obtain measurements

The majority of participants (93.4%, 341/365) reported feeling confident self-reporting height, weight and BP, while 6.6% (24/365) did not. Of 365 participants, 327 provided data on their process of BP measurement that made classification of clinical appropriateness possible, and 90.5% of these (296/327) reported processes that were clinically appropriate (supplementary material A).

Half of participants (50.1%, 181/361) measured their weight at home, 31.0% (112/361) reported a measurement from a clinic, 3.3% (12/361) from a pharmacy, 14.7% (53/361) from non-clinical setting, such workplace, gym or university, and 0.8% (3/361) participants reported estimating their weight measurements.

3.3. Agreement between BP and BMI readings

Based on researcher- measured figures, the prevalence of UKMEC 3&4 BMI and BP was 1.4% (5/356) and 3.0% (11/365) respectively. Kappa statistics suggest that the agreement between self-reported and researcher-measured BMI data was substantial (0.72 [95% confidence intervals [CI] 0.42, 1.00]), but agreement between self-reported and researcher-measured BP was poor (0.06 [95% CI -0.11, 0.23]). Two-by-two tables from which agreement was derived are presented in supplementary material B and C, full results are presented in table 2. Self-reported height and weight readings identified 80.0% (95% CI 28.4 – 99.5) of individuals with a researcher-measured BMI corresponding to UKMEC 3 or 4. Self-reported BP readings identified 9.1% (95% CI 28.4 – 99.5) of those with a researcher-measured BP corresponding to UKMEC 3 or 4. The specificity of self-reported BMI and BP measurements was high at 99.4% (95% CI 98.0- 99.9), and 97.2% (95% CI 94.9 – 98.6) respectively. Of the ten participants who were found to have self-reported BP UKMEC 1 or 2, but measured as a 3 or 4, eight reported clinically appropriate BP measurement processes and two reported unclassifiable processes.

Table 2: Agreement between self-reported and researcher-measured values

For peer review only - http://bmjopen.bmj.com/site/about/guidelines.xhtml

	N	Prevalence of UKMEC 3&4 (n, %)	Sensitivity % (95% CI)	Specificity % (95% CI)	Positive predictive value % (95% CI)	Negative predictive value % (95% CI)	Cohen's Kappa coefficient	
							Kappa coefficient (95% CI) *	P value
**UKMEC categorisation for BMI (1&2 vs 3&4)**	365	5 (1.4%)	80.0 (28.4, 99.5) %	99.4 (98.0, 99.9) %	66.7 (22.3, 95.7) %	99.7 (98.5, 100.00) %	0.72 (0.42, 1.00)	0.0000
**Blood pressure (<140/90 vs > or= 140/90)**	365	11 (3.0%)	9.1 (0.23, 41.3) %	97.2 (94.9, 98.6) %	9.1 (0.23, 41.3) %	97.2 (94.9, 98.6) %	0.06 (-0.11, 0.23)	0.1156

Self-reported weight was on average 1.8 kg lower (95% CI -2.4, -1.2) and the resulting BMI on average 0.70 kg/cm^2 lower (95% CI -0.94, -0.47) than researcher-measured readings. Systolic BP was 8mmHg higher (95% CI 7.1, 9.6) in the self-reported data than researcher-measured data. Average differences for height were <1mm (95% CI 0.0002, 0.008) and for diastolic BP were <1mmHg (95% CI -0.54, 1.44) (see table 3).

Table 3: Mean differences between self-reported and measured values; Pearson's correlation coefficient and Bland Altman limits of agreement

	Mean (SD)		Mean difference (95% CI); SD	Pearson's correlation coefficient	Bland Altman limits of agreement
	Self-reported values	Measured values			
**Height (m)**	1.65 (0.07)	1.65 (0.06)	0.004 (0.000, 0.008); 0.038	0.844	-
**Weight (kg)**	62.7 (10.4)	64.5 (12.4)	-1.8 [a] (-2.4, -1.2); 5.8	0.883	-
**Body mass index (kg/m2)**	22.9 (3.6)	23.6 (4.1)	-0.70 [a] (-0.94, -0.47); 2.3	0.832	- 5.2, 3.8
**Systolic blood pressure (mmHg)**	115.8 (10.4)	107.5 (9.7)	8.36 [a] (7.1, 9.6); 11.9	0.300	-15.4 to 32.1
**Diastolic blood pressure (mmHg)**	74.8 (8.5)	74.3 (7.4)	0.45 (-0.54, 1.4); 9.6	0.276	-18.8, 19.7

[a] $P < 0.05$, paired t test, difference between self-reported and measured values versus Ho: mean(diff) =0

Bland Altman plots (figures 2-4) for BMI and systolic and diastolic BP illustrate the degree of agreement between self-reported and researched-measured values (17). Normal distribution of the differences was verified with a histogram. Limits of agreement are wider in systolic and diastolic BP than in BMI (also see table 3). The plots show that for both BP and BMI there was no trend in differences between measurements; participants with both a high and low mean BP or BMI from self-reported and researcher measurements, were found to have variable differences in self-reported and measured BP or BMI.

4. Discussion

4.1. Key findings

For peer review only - http://bmjopen.bmj.com/site/about/guidelines.xhtml

For peer review only

Self-reported BMI was found to have substantial agreement with researcher-measured BMI, unlike self-reported BP, which had poor agreement. The majority of participants undertook a clinically acceptable process in obtaining self-reported BP. On average, participants slightly under-reported their weight and over-reported their systolic BP. Bland Altman plots showed better agreement for BMI than for systolic and diastolic BP. The plots also showed no trends or systemic variation in differences between self-reported and researcher measurements.

4.2. Where this sits within the literature

Research from Australia, Hong Kong, Finland, Malaysia, Sweden and the United States has found similar, substantial agreement for self-reported and researcher/clinician-measured weight and height for populations of all ages (18–23). Similar to our findings, in other studies which compared both self-reported and researcher-measured BP and BMI, agreement was stronger between BMI measures than between BP measurements (21,24). Studies comparing self-reported and researcher/clinician-measured BP have found varying levels of agreement, with higher agreement often found older populations (24–26). A large cohort study (n=1537) in New Zealand found kappa between self-reported and measured BP in 18-44 year olds to be 0.26 ($p < 0.001$), with a sensitivity of 18.1% and a specificity of 99.1% (25). Within the same study, those aged 45-64 had an agreement of 0.49 ($p < 0.001$), a sensitivity of 47.5%. A smaller study (n=200) of Italian women in their 50s found a kappa of 0.319 for self-reported compared with measured hypertension (24). Self-measurement of BP is more accurate than clinician-measured BP for those who are hypertensive, at risk of hypertension or are pregnant, possibly because they receive support and training for correct measurement (27–29). Self-monitoring of BP is becoming a common activity for some of these patient groups, for example amongst both hypertensive and non-hypertensive pregnant women, and those who self-monitor may have better knowledge about their BP (30).

Findings from a nested qualitative sample within this study reflect a difference in self-reporting BMI and BP experiences, where height and weight measurements were accessible and familiar but BP readings were not. The qualitative study showed that participants worked hard to understand and measure BP through a combination of recent/past measurements, borrowed machines, health service visits and online research. This analysis suggests the importance of the acknowledgement of work required to measure BP, evidence of credible human support and a digital interface that communicates the health benefits of accurate measurement (15).

4.3. Strengths and limitations

For peer review only - http://bmjopen.bmj.com/site/about/guidelines.xhtml

This is the first study of its kind to observe self-reporting of BP and BMI to obtain the COC in a young, healthy population of reproductive age. It is timely as innovative, self-care interventions for SRH become more prevalent (31,32).

We have compared self-reported and researcher-measured BP. Although, prior to contraception provision, clinicians taking single measures of blood pressure is standard, the assumption that the measurement from the research visit reflects the true BP is problematic. BP is inherently variable, influenced by numerous extrinsic and intrinsic factors (33). A systematic review of the sources of inaccuracy in the measurement of adult resting BP, identified significant directional effects from 27 sources, resulting in a range of -23.6 to +33.0 mmHg for systolic BP and -14.0 to +23.0 mmHg for diastolic BP (34). Whilst researchers employed recommended strategies to mitigate inaccuracies in BP measurement, some factors remain irremediable, for example 'white coat syndrome', whereby researcher-, or clinician-, measured BP are artificially raised due to anxiety, or natural BP variation (35,36). Recently updated UK guidance recommends ambulatory or home BP monitoring involving multiple repeated measures over a day for ambulatory or at least 4 days for home BP monitoring to diagnose hypertension (13).

There was potential for self-selection bias during recruitment where users who may have estimated their BP during their order might have been unwilling to participate. To mitigate this, invitation communication, whilst providing sufficient information on the study procedure, did not emphasise validation of measurements (see supplementary material D). The study had good uptake, with 44.1% of eligible users attending a research visit, where researchers reiterated their independence from the online service.

Almost all study participants had experience of taking COC before ordering from the online service (96.2%, 351/365), a figure that is greater than amongst non-responders (91.1%, 428/470, $p<0.01$). This suggests potential healthy user bias, in that those with more experience of COC may be more likely to agree to participate. Such experienced COC users may be less likely to have high BP or BMI, and could be more familiar with, or more aware of the importance of, strategies to obtain biometric readings for remote prescription that might be clinically appropriate.

The medium time between COC order and research visit was 20 days. This may have impacted on participants' recall of the details of recent BP measurements. This is evident in the 38 unclassifiable participant processes: 31 participants gave the date of their most recent BP measurement between their order and visit, seven were unclassifiable due to limited information in their response.

For peer review only - http://bmjopen.bmj.com/site/about/guidelines.xhtml

There are methodological limitations that may explain the low kappa coefficient. The kappa produced, in particular for BP, should be interpreted considering the method's paradoxes and problems. The two-by-two table for BP (supplementary material C) produced, with it's perfectly symmetrically unbalanced marginal totals, satisfies both paradoxes outlined by Feinstein and Cicchetti, which, in addition to the low prevalence of researcher-measured high BMI and BP, perhaps provides an additional factor explaining the low kappa in light of high percentage agreement (37).

4.4. Meaning and mechanism

Explanations for our findings include the natural variability of BP, white coat syndrome, or inaccurate self-measurement or reporting (35,36). Although our quantitative data does not allow us to explore whether our findings are due to the natural variability of BP or to inaccurate self-measurement or reporting, our qualitative research provides some insights (15). Participants shared both experiences of white coat syndrome at previous consultations, and, in relation to their online COC order, difficulties obtaining a new BP measurement, remembering values from a recent reading, and difficulties interpreting or uploading readings, as well as a single participant who recounted misreporting despite a known high BP.

Whilst BP limits of agreement are comparable to biologically plausible ranges established when estimating the impact of sources of inaccuracy (34), the clinically relevant factor for remote COC prescription is not the magnitude of these limits, but whether they shift BP from a non-contraindication to a contraindication. Nevertheless, at their widest, such limits would trigger clinical concern and might initiate a conversation about process and accuracy of measurement.

The difference in findings for BP and BMI might reflect differences in familiarity with their measurement. Fifty years ago, accurate equipment for measuring weight were rare in households and most people did not know their current weight. BP monitors are now transitioning from a technology predominantly available in health care settings to one that is increasingly available at home. Strategies to increase accuracy of reporting could focus on improving understanding of BP and access to measurement.

5. Implications and recommendations for online contraceptive services

Our findings, understood in the context of findings from the nested qualitative research (15), have implications for online contraceptive services, including the service evaluated in this study. In response to the findings, in collaboration with the study team, SH:24 have completed a process of user-centred design that aims to create a 'safe and supportive enabling environment' to maximise the benefits and minimise the risks of self-care, acknowledging the responsibility of service providers

For peer review only - http://bmjopen.bmj.com/site/about/guidelines.xhtml

in this respect (31,38) The design process suggested innovations that include: building skills for self-monitoring in face-to-face care when BP is measured by clinicians; providing home BP monitors with support to use them; improved online information on the importance of BP measurements and consequences of inaccurate reporting; credible clinical support as required; issuing a short supply for the first order with information on how to obtain a BP measurement (15,39).These strategies may improve the accuracy of BP reporting and we recommend further research to evaluate them.

6. Conclusions

Online contraceptive services provide a convenient alternative for oral contraception users which may improve uptake and continuation (5). Accuracy of self-reported height, weight and BP is important to ensure appropriate, safe COC prescription online, since BMI and BP may contraindicate use (6,7).

Whilst self-reported BMI was found to have substantial agreement with researcher-measured BMI, self-reported BP was shown to have poor agreement with researcher-measured BP. These differences may be due to the natural variability of BP, white coat syndrome, methodological limitations associated with kappa or by inaccurate self-measurement and reporting.

As online contraceptive services are increasingly prevalent and self-measured BP has been shown to be valid in other populations, strategies to improve BP self-measurement for remote prescription of COC are needed.

Acknowledgements

We thank all users of the online service who took part in this study. We thank SH:24 for collaborating on the project, in particular the clinical team, designers and data analyst at the online service for their support with recruitment and data collection.

Ethical considerations

The East Midlands – Leicester Research Ethics Committee granted ethical approval to the project (Evaluation of online provision of oral contraceptives to measure: accuracy of self-reported height, weight and blood pressure; essential information transfer and user experience, reference 17/EM/0181) on 6th June 2017. Before participating in a research visit, users were presented with a participant information sheet, were given the opportunity to ask questions, which they had answered to their satisfaction, and then provided informed consent. Participation was voluntary, and participants were assured that the information they provided would be anonymised and kept confidential. All participants whose biometric data suggested a UKMEC category 3 or 4 or who

For peer review only

reported contraindications to the COC during the study or indicated a need for further clinical support were referred to a local sexual health service.

Funding

This report is independent research funded by the National Institute for Health Research (Research for Patient Benefit Programme, PB-PG-0815-20009). The views expressed in this publication are those of the author(s) and not necessarily those of the NIHR or the Department for Health and Social Care. The sponsor, King's College Hospital, was not involved in study design, data collection, analysis, or interpretation of data.

Data statement

The anonymised dataset is available from the authors on request.

Competing interests

Since completion of this study, HM has worked at the online service on a different research project. PB is clinical director of the online service studied and is one of the prescribers within this service. AM has also been employed by the online service as a bank clinical support midwife. This work is part of a process of research on innovation at SH:24 where all innovation is the subject of research to ensure that learning is shared widely. SH:24 is a 'not for profit' organisation that provides health services to the National Health Service and sharing learning through innovation and research is one of the principles of the organisation.

Author contributions

Hannah McCulloch contributed to data collection, led the data analysis, and drafted the manuscript.

Alessandra Morelli contributed to data collection and data analysis and commented on the manuscript.

Caroline Free contributed to the conception and design of the study, contributed to data analysis and commented on the manuscript.

Jonathan Syred contributed to the conception and design of the study, contributed to data collection and commented on the manuscript.

Riley Botelle contributed to data collection and commented on the manuscript.

Paula Baraitser contributed to the conception and design of the study, oversaw data collection, contributed to data analysis and commented on the manuscript.

For peer review only

For peer review only - http://bmjopen.bmj.com/site/about/guidelines.xhtml

Figure headings

Figure 1: Recruitment Flow Chart for users of the online service

Figure 2. Bland Altman plot of the differences between self-reported and researcher-measured BMI compared with the mean self-reported and researcher-measured BMI. The central line represents the mean difference between self-reported and researcher-measured BMI. The upper and lower boundaries represent the 95% limits of agreement.

Figure 3: Bland Altman plot of the differences between self-reported and researcher-measured systolic BP compared with the mean self-reported and researcher-measured systolic BP. The central line represents the mean difference between self-reported and researcher-measured systolic BP. The upper and lower boundaries represent the 95% limits of agreement.

Figure 4. Bland Altman plot of the differences between self-reported and researcher-measured diastolic BP compared with the mean self-reported and researcher-measured diastolic BP. The central line represents the mean difference between self-reported and researcher-measured diastolic BP. The upper and lower boundaries represent the 95% limits of agreement.

References

1. French RS, Gibson L, Geary R, Glasier A, Wellings K. Changes in the prevalence and profile of users of contraception in Britain 2000–2010: evidence from two National Surveys of Sexual Attitudes and Lifestyles. BMJ Sexual & Reproductive Health [Internet]. 2020 Jan 21 [cited 2020 Jan 29];bmjsrh-2019-200474. Available from: http://jfprhc.bmj.com/lookup/doi/10.1136/bmjsrh-2019-200474

2. Royal College of Obstetricians and Gynaecologists. Better for Women: Improving the health and wellbeing of girls and women [Internet]. 2019. Available from: https://www.rcog.org.uk/en/news/campaigns-and-opinions/better-for-women/

3. Wilson E, Baraitser P, Free C. Does internet-accessed STI (e-STI) testing increase testing uptake for chlamydia and other STIs among a young population who have never tested? Secondary analyses of data from a randomised controlled trial. Sex Transm Infect [Internet]. 2019 [cited 2019 Jul 15];0:1–6. Available from: http://sti.bmj.com/

4. Wilson E, Free C, Morris T, Syred J, Ahamed I, Menon-Johansson A, et al. Internet-accessed sexually transmitted infection (e-STI) testing and results service: A randomised, single-blind, controlled trial. PLoS ONE. 2017;14(12).

5. Rezel-Potts E, Free C, Syred J, Baraitser P. Expanding choice through online contraception: A theory of change to inform service development and evaluation. BMJ Sexual and Reproductive Health. 2019;

6. Faculty of Sexual and Reproductive Healthcare. UK Medical eligibility criteria for contraceptive use: UKMEC 2016 (Updated 2019).

For peer review only

For peer review only - http://bmjopen.bmj.com/site/about/guidelines.xhtml

7. World Health Organisation. Medical Eligibility Criteria for Contraceptive Use [Internet]. 2015. Available from: https://www.who.int/reproductivehealth/publications/family_planning/Ex-Summ-MEC-5/en/

8. SH:24. Programme overview [Internet]. Available from: https://sh24.org.uk/

9. Public Health England. Sexual and Reproductive Health Profiles [Internet]. 2020 [cited 2020 Mar 4]. Available from: https://fingertips.phe.org.uk/profile/sexualhealth/data#page/0/gid/8000057/pat/6/par/E12000007/ati/102/are/E09000028

10. Lambeth Southwark and Lewisham Public Health Departments. Lambeth, Southwark and Lewisham Sexual and Reproductive Health Strategy 2019–24 [Internet]. London; 2019. Available from: https://www.lambeth.gov.uk/sites/default/files/yh-lsl-sexual-health-strategy_0.pdf

11. General Medical Council. Good practice in prescribing and managing medicines and devices [Internet]. 2013. Available from: https://www.gmc-uk.org/Prescribing_guidance.pdf_59055247.pdf

12. Topouchian J, Agnoletti D, Blacher J, Youssef A, Chahine MN, Ibanez I, et al. Validation of four devices: Omron M6 Comfort, Omron HEM-7420, Withings BP-800, and Polygreen KP-7670 for home blood pressure measurement according to the European society of hypertension international protocol. Vascular Health and Risk Management. 2014;10:33–44.

13. National Institute for Health and Care Excellence. Hypertension in adults: diagnosis and management [Internet]. Available from: https://www.nice.org.uk/guidance/ng136

14. Sim J, Wright CC. The Kappa Statistic in Reliability Studies: Use, Interpretation, and Sample Size Requirements. Physical Therapy [Internet]. 2005 Mar 1 [cited 2020 Jan 31];85(3):257–68. Available from: https://academic.oup.com/ptj/article/85/3/257/2805022

15. Baraitser P, McCulloch H, Morelli A, Free C. How do users of a digital-only contraceptive service provide biometric measurements and what does this teach us about safe and effective online care? A qualitative interview study. BMJ Open. 2020;10(9).

16. Faculty of Sexual & Reproductive Healthcare (FSRH). Collaboration, Choice, Care: The Contraception Priority Setting Partnership (PSP) [Internet]. 2018 [cited 2021 Jun 29]. Available from: https://www.fsrh.org/documents/fsrh-contraception-psp-report-2018-jla/

17. Giavarina D. Understanding Bland Altman analysis. Biochemia Medica. 2015;25(2):141–51.

18. Davies A, Wellard-Cole L, Rangan A, Allman-Farinelli M. Validity of self-reported weight and height for BMI classification: A cross-sectional study among young adults. Nutrition. 2020 Mar 1;71:110622.

19. Dahl A, Hassing L, Fransson E, Pederson N. Agreement between self-reported and measured height, weight and body mass index in old age—a longitudinal study with 20 years of follow-up. Age and Ageing [Internet]. 2010 [cited 2020 Feb 27];39(4):445–51. Available from: https://www.ncbi.nlm.nih.gov/pmc/articles/PMC2899942/

20. Tuomela J, Kaprio J, Sipilä PN, Silventoinen K, Wang X, Ollikainen M, et al. Accuracy of self-reported anthropometric measures — Findings from the Finnish Twin Study. Obesity Research and Clinical Practice. 2019 Nov 1;13(6):522–8.

For peer review only - http://bmjopen.bmj.com/site/about/guidelines.xhtml

21. Murphy G, Snetselaar L, Myers E, LaForge B, Qualls LG, Hand RK, et al. Self-reported health parameters compared with clinician measurements: methods in practice-based research. Journal of public health management and practice : JPHMP [Internet]. 2014 [cited 2022 Jan 10];20(5):513–22. Available from: https://pubmed.ncbi.nlm.nih.gov/24352004/

22. Olfert MD, Barr ML, Charlier CM, Famodu OA, Zhou W, Mathews AE, et al. Self-Reported vs. Measured Height, Weight, and BMI in Young Adults. International journal of environmental research and public health [Internet]. 2018 Oct 11 [cited 2022 Jan 10];15(10). Available from: https://pubmed.ncbi.nlm.nih.gov/30314261/

23. Kee CC, Lim KH, Sumarni MG, Teh CH, Chan YY, Nuur Hafizah MI, et al. Validity of self-reported weight and height: a cross-sectional study among Malaysian adolescents. BMC medical research methodology [Internet]. 2017 Jun 2 [cited 2022 Jan 10];17(1). Available from: https://pubmed.ncbi.nlm.nih.gov/28577547/

24. Villarini M, Acito M, Gianfredi V, Berrino F, Gargano G, Somaini M, et al. Validation of Self-Reported Anthropometric Measures and Body Mass Index in a Subcohort of the DianaWeb Population Study. Clinical Breast Cancer [Internet]. 2019 Aug 1 [cited 2020 Feb 20];19(4):e511–8. Available from: http://www.ncbi.nlm.nih.gov/pubmed/31182401

25. Taylor A, Grande ED, Gill T, Pickering S, Grant J, Adams R, et al. Comparing self-reported and measured high blood pressure and high cholesterol status using data from a large representative cohort study. Australian and New Zealand Journal of Public Health [Internet]. 2010 Aug 1 [cited 2020 Feb 20];34(4):394–400. Available from: http://doi.wiley.com/10.1111/j.1753-6405.2010.00572.x

26. Ning M, Zhang Q, Yang M. Comparison of self-reported and biomedical data on hypertension and diabetes: Findings from the China Health and Retirement Longitudinal Study (CHARLS). BMJ Open. 2016;6(1).

27. McManus RJ, Wood S, Bray EP, Glasziou P, Hayen A, Heneghan C, et al. Self-monitoring in hypertension: A web-based survey of primary care physicians. Journal of Human Hypertension. 2014;28(2):123–7.

28. Hinton L, Tucker KL, Greenfield SM, Hodgkinson JA, Mackillop L, McCourt C, et al. Blood pressure self-monitoring in pregnancy (BuMP) feasibility study; A qualitative analysis of women's experiences of self-monitoring. BMC Pregnancy and Childbirth. 2017 Dec 19;17(1).

29. Tucker KL, Taylor KS, Crawford C, Hodgkinson JA, Bankhead C, Carver T, et al. Blood pressure self-monitoring in pregnancy: Examining feasibility in a prospective cohort study. BMC Pregnancy and Childbirth. 2017 Dec 28;17(1).

30. Tucker KL, Hodgkinson J, Wilson HM, Crawford C, Stevens R, Lay-Flurrie S, et al. Current prevalence of self-monitoring of blood pressure during pregnancy: the BUMP Survey. Journal of hypertension [Internet]. 2021 May 1 [cited 2022 Jan 10];39(5):994–1001. Available from: https://pubmed.ncbi.nlm.nih.gov/33399304/

31. Narasimhan M, Allotey P, Hardon A. Self care interventions to advance health and wellbeing: A conceptual framework to inform normative guidance. BMJ (Online). 2019;365.

32. World Health Organization (WHO). WHO Consolidated Guideline on Self-Care Interventions for Health: Sexual and Reproductive Health and Rights. 2020; Available from: https://apps.who.int/iris/bitstream/handle/10665/325480/9789241550550-eng.pdf?ua=1

For peer review only

For peer review only - http://bmjopen.bmj.com/site/about/guidelines.xhtml

33. Pickering TG. Blood pressure variability and ambulatory monitoring. [Internet]. Vol. 2, Current opinion in nephrology and hypertension. 1993 [cited 2020 Mar 2]. p. 380–5. Available from: http://www.ncbi.nlm.nih.gov/pubmed/7922198

34. Kallioinen N, Hill A, Horswill MS, Ward HE, Watson MO. Sources of inaccuracy in the measurement of adult patients' resting blood pressure in clinical settings: A systematic review. Vol. 35, Journal of Hypertension. Lippincott Williams and Wilkins; 2017. p. 421–41.

35. McGrath BP. Ambulatory blood pressure monitoring and "white coat" hypertension: Saving costs. Vol. 176, Medical Journal of Australia. 2002. p. 571–2.

36. Turner MJ, van Schalkwyk JM. Blood pressure variability causes spurious identification of hypertension in clinical studies: A computer simulation study. American Journal of Hypertension. 2008 Jan;21(1):85–91.

37. Feinstein AR, Cicchetti D v. High agreement but low Kappa: I. the problems of two paradoxes. Journal of Clinical Epidemiology. 1990;43(6):543–9.

38. Ferguson L, Fried S, Matsaseng T, Ravindran S, Gruskin S. Human rights and legal dimensions of self care interventions for sexual and reproductive health. The BMJ. 2019;365.

39. Baraitser P, McCulloch H, Shather Z, Free C. Supporting blood pressure measurement prior to online prescription of the combined oral contraceptive. BMJ Sexual and Reproductive Health. 2021;

For peer review only

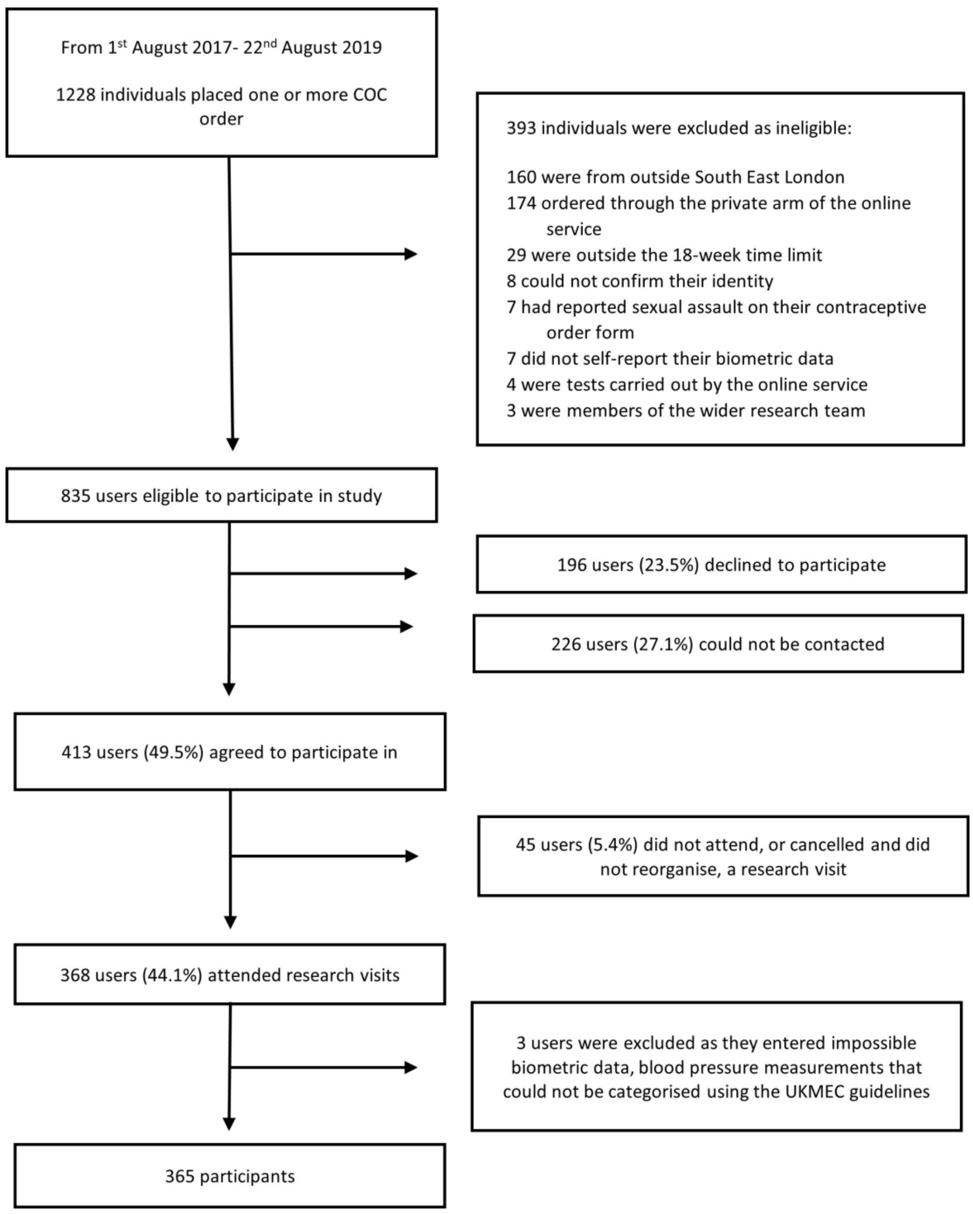

Figure 1: Recruitment Flow Chart for users of the online service

154x192mm (330 x 330 DPI)

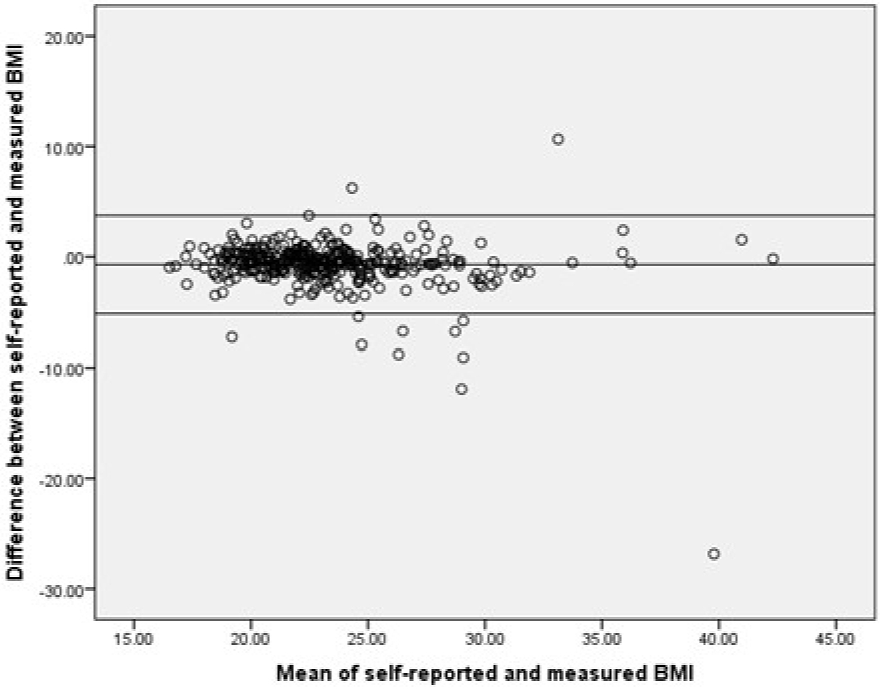

Figure 2. Bland Altman plot of the differences between self-reported and researcher-measured BMI compared with the mean self-reported and researcher-measured BMI. The central line represents the mean difference between self-reported and researcher-measured BMI. The upper and lower boundaries represent the 95% limits of agreement.

119x93mm (96 x 96 DPI)

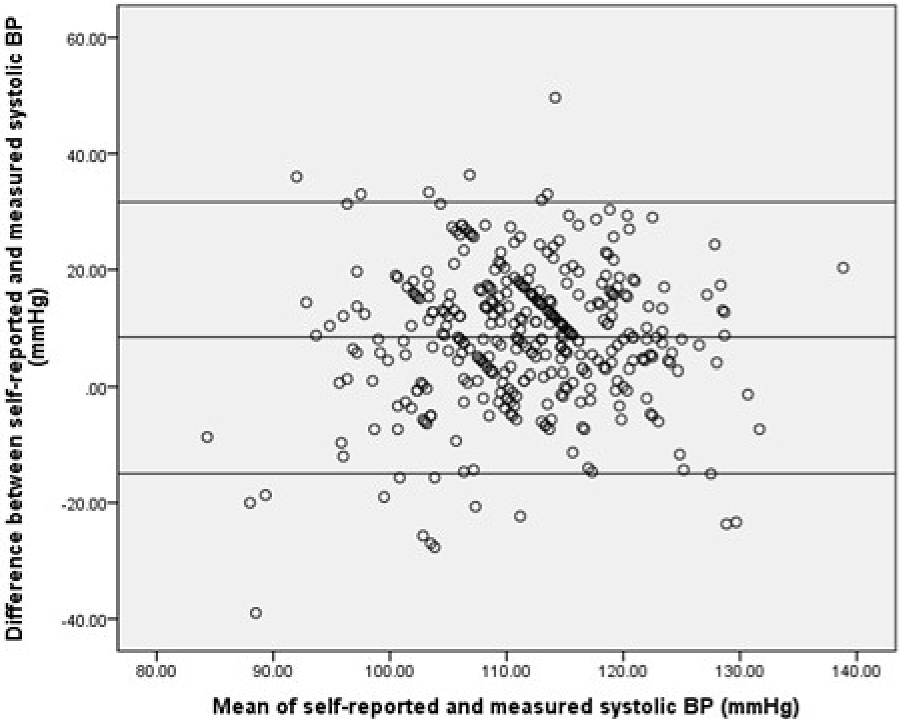

Figure 3: Bland Altman plot of the differences between self-reported and researcher-measured systolic BP compared with the mean self-reported and researcher-measured systolic BP. The central line represents the mean difference between self-reported and researcher-measured systolic BP. The upper and lower boundaries represent the 95% limits of agreement

122x97mm (96 x 96 DPI)

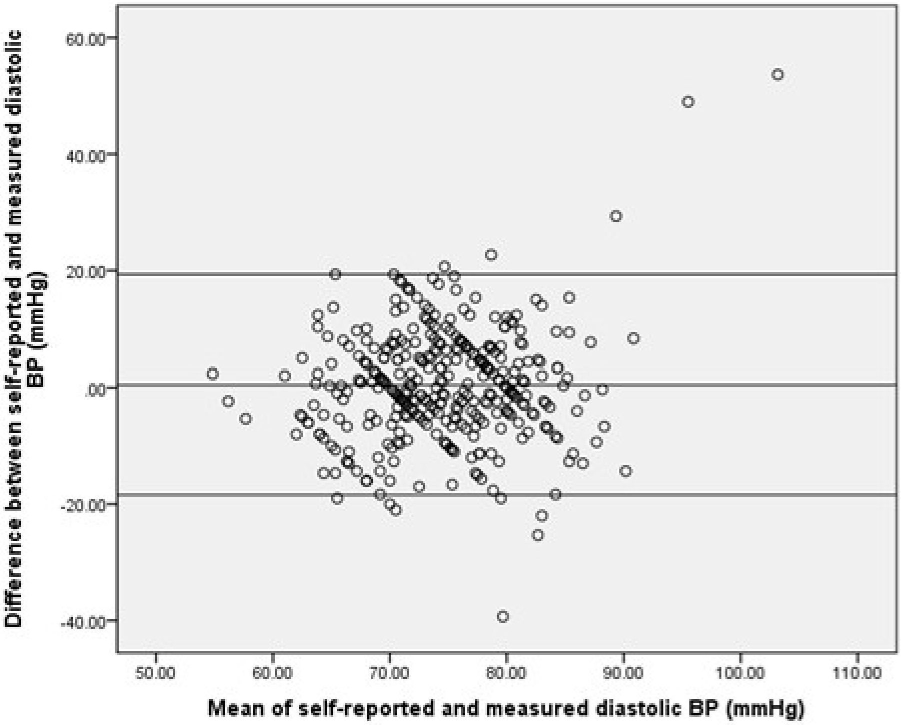

Figure 4. Bland Altman plot of the differences between self-reported and researcher-measured diastolic BP compared with the mean self-reported and researcher-measured diastolic BP. The central line represents the mean difference between self-reported and researcher-measured diastolic BP. The upper and lower boundaries represent the 95% limits of agreement.

122x98mm (96 x 96 DPI)

Supplementary material

A. Process of BP measurement and examples from free text answers

Process of blood pressure measurement	Example
Unclassifiable	- I have a blood pressure monitor at home (date given between order and research visit)<br>- "My blood pressure was measured by a colleague at work (12 days before order)<br>- "[South East London Clinic] (date given between order and research visit)"<br>- "I have a nurse at my office that offer blood pressure testing so I used the facilities provided to check (date given between order and research visit)"<br>- "I visited the pharmacy stand in boots. They took me to a private room and took my blood pressure for me (date given between order and research visit)"
Clinically appropriate	- "From my GP when I was first prescribed the pill (3 months before order)"<br>- "The nurse in the clinic took my blood pressure (same day as order)"<br>- "Inherited blood pressure machine from relative with long term illness who no longer needed it. Used this at home to obtain measure (4 days before order)<br>- "I visited my aunts house to get my blood pressure reading since she has a machine at home (day before order)<br>- "I went to my local pharmacy where they had a self checker machine that calculated my blood pressure, weight and height (same day as order)"
Clinically inappropriate	- "At the GP before getting a repeat prescription of the pill, which was measured normally (18 months before order)"<br>- "A bupa nurse came into my place of work and offered a health check. My blood pressure was taken there (2 years before order)<br>- "I looked up what a healthy blood pressure would be on NHS website and put in that blood pressure when on the sh:24 website"<br>- "Estimated. Had a blood pressure machine but feel I did not use it correctly"<br>- "Googled healthy blood pressure"

For peer review only - http://bmjopen.bmj.com/site/about/guidelines.xhtml

B. Self-reported and researcher-measured BMI

		Measured BMI		
		UKMEC 1&2 <35.0 kg/m	UKMEC 3&4 > or= 35 kg/m	Total
Self-reported BMI	UKMEC 1&2 <35.0 kg/m	358	1	359
	UKMEC 3&4 > or= 35 kg/m	2	4	6
	Total	360	5	365

C. Self-reported and researcher-measured BP

		Researcher-measured blood pressure (mmHg)		
		< 140/90	> or = 140/90	Total
Self-reported blood pressure (mmHg)	<140/90	344	10	354
	> or= 140/90	10	1	11
	Total	354	11	365

D. SMS invitation message

“Hello. SH:24 are working with King's College Hospital to improve contraception services. King's would like to invite you to take part in their research - they will thank you for your time with £45. The research would involve meeting a member of the King's team to allow them to check your height, weight and blood pressure. You will also be asked to complete a short questionnaire (this should take no more than 40 mins in total). Text back NO if you don't want to receive a call back from the King's research team. Thanks, SH:24"

For peer review only - http://bmjopen.bmj.com/site/about/guidelines.xhtml

Agreement between self-reported and researcher-measured height, weight and blood pressure measurements for online prescription of the combined oral contraceptive pill: an observational study

Journal:	*BMJ Open*
Manuscript ID	bmjopen-2021-054981.R2
Article Type:	Original research
Date Submitted by the Author:	29-Mar-2022

Complete List of Authors:	McCulloch, Hannah; King's College London Faculty of Life Sciences and Medicine, King's Centre for Global Health and Health Partnerships<br>Morelli, Alessandra; King's College London Faculty of Life Sciences and Medicine, King's Centre for Global Health and Health Partnerships<br>Free, Caroline; London School of Hygiene and Tropical Medicine Faculty of Epidemiology and Population Health, Department of Population Health<br>Syred, Jonathan; King's College London Faculty of Life Sciences and Medicine, King's Centre for Global Health and Health Partnerships<br>Botelle, Riley; King's College London Faculty of Life Sciences and Medicine, Kings Centre for Global Health and Health Partnerships<br>Baraitser, Paula; King's College Hospital, Sexual Health; SH:24 CIC

**Primary Subject Heading**:	Reproductive medicine
Secondary Subject Heading:	Public health, Sexual health
Keywords:	SEXUAL MEDICINE, REPRODUCTIVE MEDICINE, PUBLIC HEALTH

SCHOLARONE™
Manuscripts

I, the Submitting Author has the right to grant and does grant on behalf of all authors of the Work (as defined in the below author licence), an exclusive licence and/or a non-exclusive licence for contributions from authors who are: i) UK Crown employees; ii) where BMJ has agreed a CC-BY licence shall apply, and/or iii) in accordance with the terms applicable for US Federal Government officers or employees acting as part of their official duties; on a worldwide, perpetual, irrevocable, royalty-free basis to BMJ Publishing Group Ltd ("BMJ") its licensees and where the relevant Journal is co-owned by BMJ to the co-owners of the Journal, to publish the Work in this journal and any other BMJ products and to exploit all rights, as set out in our licence.

The Submitting Author accepts and understands that any supply made under these terms is made by BMJ to the Submitting Author unless you are acting as an employee on behalf of your employer or a postgraduate student of an affiliated institution which is paying any applicable article publishing charge ("APC") for Open Access articles. Where the Submitting Author wishes to make the Work available on an Open Access basis (and intends to pay the relevant APC), the terms of reuse of such Open Access shall be governed by a Creative Commons licence – details of these licences and which Creative Commons licence will apply to this Work are set out in our licence referred to above.

Other than as permitted in any relevant BMJ Author's Self Archiving Policies, I confirm this Work has not been accepted for publication elsewhere, is not being considered for publication elsewhere and does not duplicate material already published. I confirm all authors consent to publication of this Work and authorise the granting of this licence.

For peer review only

Agreement between self-reported and researcher-measured height, weight and blood pressure measurements for online prescription of the combined oral contraceptive pill: an observational study

Hannah McCulloch[1], Alessandra Morelli[1], Caroline Free[2], Jonathan Syred[1], Riley Botelle[1], Paula Baraitser[134]

Author affiliations

[1] King's College London, King's Centre for Global Health and Health Partnerships, London, United Kingdom

[2] London School of Hygiene and Tropical Medicine, Department of Population Health, London, United Kingdom

[3] King's College Hospital, Camberwell Sexual Health Clinic, 94-104 Denmark Hill, SE5 9RS, London, United Kingdom

[4] SH:24, 35A Westminster Bridge Road, SE1 7JB, London, United Kingdom

Corresponding author:

Hannah McCulloch, King's College London, King's Centre for Global Health and Health Partnerships, Weston Education Centre, 10 Cutcombe Rd, SE5 9RJ London, United Kingdom; hrmcculloch@gmail.com

Title word count: 23

Abstract word count: 262

Strengths and limitations statement: 121

Full article word count: 2967

For peer review only

For peer review only - http://bmjopen.bmj.com/site/about/guidelines.xhtml

Abstract

Title: Agreement between self-reported and researcher-measured height, weight and blood pressure measurements for online prescription of the combined oral contraceptive pill: an observational study

Objective(s): To compare agreement between self-reported height, weight and blood pressure measurements submitted to an online contraceptive service with researcher-measured values and document strategies used for self-reporting.

Design: An observational study.

Setting: An online sexual health service which provided the combined oral contraceptive pill, free of charge, to users in Southeast London, England.

Participants: Between August 2017 and August 2019, 365 participants were recruited.

Primary and secondary outcome measures: The primary outcome, for which the study was powered, was the agreement between self-reported and researcher-measured body mass index and blood pressure measurements, compared using kappa coefficients. Secondary measures of agreement included sensitivity, specificity, and Bland Altman plots. The study also describes strategies used for self-reporting and classifies their clinical appropriateness.

Results: 327 participants fully described their process of blood pressure measurement with 296 (90.5%) classified as clinically appropriate. Agreement between self-reported and researcher-measured body mass index was substantial (0.72 [95% confidence intervals [CI] 0.42, 1.0]), but poor for blood pressure (0.06 [95% CI -0.11, 0.23]). Self-reported height and weight readings identified 80.0% (95% CI 28.4 - 99.5) of individuals with a researcher-measured high body mass index (≥ than 35kg/m2) and 9.1% (95% CI 0.23, 41.3) of participants with a researcher-measured high blood pressure (≥ 140/90 mmHg).

Conclusion: In this study, whilst self-reported BMI was found to have substantial agreement with researcher-measured BMI, self-reported BP was shown to have poor agreement with researcher-measured BP. This may be due to the inherent variability of blood pressure, overdiagnosis of hypertension by researchers due to 'white coat syndrome' or inaccurate self-reporting. Strategies to improve self-reporting of blood pressure for remote prescription of the combined pill are needed.

Strengths and limitations of this study

- This is the first study to observe self-reporting of blood pressure and body mass index to obtain the combined oral contraceptive pill in a healthy population of reproductive age.

For peer review only - http://bmjopen.bmj.com/site/about/guidelines.xhtml

- We compared blood pressure and body mass index measurements self-reported by users online with researcher-measured readings, taken on average 20 days later.
- This study's assumption that researcher measurements reflect true blood pressure is problematic, however, clinicians taking single measures of blood pressure is standard practice in contraceptive provision
- We recognise the potential for self-selection bias in recruitment, where users who may have estimated their blood pressure during their order might have been unwilling to participate, and for healthy user effect since the majority of participants had previous experience of taking the pill

Key words: Online sexual health services, contraception, self-care, self-reported biometric data, remote prescribing, blood pressure

For peer review only

__**Full article**__

1. Introduction

In the United Kingdom (UK), 29.5% of sexually active women of reproductive age use oral contraceptives (1). Whilst contraception is available free of cost in the UK, research has found that 37.0% of women have difficulty accessing contraceptive services (2).

For peer review only - http://bmjopen.bmj.com/site/about/guidelines.xhtml

Online sexual health services, offering tests for sexually transmitted infections (STIs) ordered online and posted home, with self-taken samples posted to laboratories and results communicated remotely, have become part of the UK sexual health economy. Online services increase access to testing, including amongst those who have never tested before (3,4).

Online services might facilitate access to contraception, enabling users to order oral contraception with remote prescribing and postal delivery (5). Remote prescription of the combined oral contraceptive (COC) requires a medical history and self-reported height, weight and blood pressure (BP). The accuracy of these biometric measurements is important to ensure appropriate, safe prescription.

The UK and World Health Organisation (WHO) medical eligibility criteria (UKMEC/WHOMEC) classify all contraceptive contraindications on a scale of 1-4 (6,7). A classification of 1 or 2 implies that benefits of COC use outweigh the risks, whilst a classification of 3 or 4 suggests that risks of use outweigh benefits. A body mass index (BMI) equal to or greater than 35kg/m2 or a BP consistently above 140/90 mmHg would be classified as UKMEC 3 or 4.

We studied a UK-based, online contraceptive service, SH:24, which provided COC via post after an online medical history and self-reported height, weight and BP (8). We aimed to compare self-reported BMI and BP measurements with measurements taken by researchers.

2. Materials and methods

2.1. Setting and ethical approval

SH:24 provided online sexual health services free to the residents of five areas of South East London, areas with poor sexual and reproductive health (SRH) indicators (9,10), between January 2017 and August 2019. SH:24 is an NHS-commissioned-service, registered with the UK Quality Care Commission and compliant with the General Medical Council guidance on remote prescribing (11). SH:24 was advertised through online media, and signposting from clinics. The study was given ethical approval by the East Midlands – Leicester Research Ethics Committee on 6th June 2017.

2.2. Data collection

Users who ordered the COC were invited to a research visit within 18 weeks of ordering. At the visit, height and weight were measured. BP was measured with the participant sitting down having rested for ten minutes, with the arm supported at heart level. Two cuff sizes were available, dependent on participant's arm circumference. Participants were asked to refrain from talking and crossing their legs during the measurements. Three readings were taken at 10-minute intervals using a clinically validated, automated sphygmomanometer (Omron M6) (12). During this research visit, participants

For peer review only

For peer review only - http://bmjopen.bmj.com/site/about/guidelines.xhtml

completed two surveys, documenting demographic information and strategies used to obtain their self-reported measurements.

2.3. Data analysis

To compare self-reported with researcher-measured values, we report Kappa coefficients, as well as sensitivity, specificity, positive predictive value (PPV) and negative predictive value (NPV), of BP and BMI. The mean of the BP readings was used as the researcher-measured reading. Self-reported and researcher-measured BPs were classified as either UKMEC 1&2 or UKMEC 3&4. We have chosen to report sensitivity, specificity, PPV and NPV of both BMI and BP, although we recognize that since data collection was completed, guidance has changed such that BP readings as measured by our researchers are no longer the 'gold standard' for diagnosing hypertension (13). Limitations of using researcher measurements as the 'true' BP value are considered the discussion.

The primary outcome, for which the study was powered, was the kappa coefficient for self-reported vs researcher-measured BMI and BP. Three hundred and sixty-five participants were needed to achieve 90% power to detect a true kappa value in a test of a null hypothesis (H0): Kappa = 0.7 vs an alternative hypothesis (H1): Kappa >/= 0.9. This was based on a significance level of 0.05 (14), and a prevalence of UKMEC 3&4 BP and BMI of 7.5%, which was estimated from contraceptive consultations at a local sexual health service in one of the study areas of South East London.

We used paired t-tests to calculate mean differences between self-reported and researcher-measured weight, height and BMI, systolic and diastolic BP readings. We report Pearson's correlation coefficients for the level of correlation and present Bland-Altman plots with mean differences and 95% limits of agreement, defined as ± 1.96 x (standard deviation of the difference). Analyses were completed using STATA (Version 15) and Bland Altman plots were created using SPSS (Version 24).

Participants were asked to describe how they obtained height, weight and BP measurements for their order. We classified these processes as clinically appropriate or inappropriate. Clinically appropriate was defined as a reading taken with a BP measurement device, or taken at a clinical location or pharmacy, or a manual reading taken by someone with the appropriate technical skill, within 12 months prior to ordering.

2.4. Public and patient involvement (PPI)

Patients and the public participated in the identification of the importance of the research question for this study and the nested qualitative study (15). Nationally significant PPI, endorsed by the UK Faculty of Sexual and Reproductive Health Care, found that among the general population 'research

For peer review only - http://bmjopen.bmj.com/site/about/guidelines.xhtml

to evaluate which interventions increase uptake and continuation rates of effective contraceptive methods' was the top priority for UK academic contraceptive research (16).Reflecting this, ahead of developing the study protocol, the research team conducted their own PPI prioritization exercise with two focus groups of contraceptive service users, which identified research into online service delivery as a priority. The logic model for the intervention was similarly produced with involvement from patients and the public including interviews with 21 stakeholders (5). Intervention development involved over 100 sexual health service users through a process of human-centred design.

3. Results

3.1. Participants

Between August 2017 and August 2019, 1228 individuals ordered COC online, of which 835 were eligible for the study and 368 (44.1%) attended a research visit (figure 1). Median time between COC order and visit was 20 days (IQR: 14-30).

For peer review only

Table 1: Sociodemographic characteristics, contraceptive and online service use of population and sample

		Eligible population n = 835, %[a]	Non-participants n = 470, %	Study participants n = 365, %	
**Sociodemographic characteristics**					
**Age (years)** [a]	**mean**	24.9	24.9	24.8	0.55[d]

18-19	86 (10.3)	57 (12.1)	29 (7.9)	0.05 [d]
20-24	360 (43.1)	185 (39.3)	175 (48.0)	
25-34	355 (42.5)	208 (44.3)	147 (40.3)	
35+	34 (4.1)	20 (4.3)	14 (8.8)	
**Ethnicity [a]**				
White English/ Welsh/ Scottish/ Northern Irish/British/ Other	524 (62.7)	303 (64.5)	221 (60.6)	0.10 [d]
Black African/ Caribbean/ British/ Other	113 (13.5)	56 (11.9)	57 (15.6)	
Asian/ Asian British	86 (10.3)	53 (11.3)	33 (9.0)	
Mixed/ multiple ethnic groups	76 (9.1)	34 (7.2)	42 (11.5)	
Other ethnic groups	31 (3.7)	20 (4.3)	10 (2.7)	
Not known/ prefer not to say	6 (0.72)	4 (0.85)	2 (0.55)	
**Index of multiple deprivation quintile [ab]**				
1 (most deprived)	282 (34.2)	151 (32.7)	131 (36.2)	0.44 [d]
2	335 (40.7)	187 (40.5)	148 (40.9)	
3	155 (18.8)	89 (19.2)	66 (18.2)	
4	42 (5.1)	29 (6.3)	13 (3.6)	
5 (least deprived)	10 (1.2)	6 (1.3)	4 (1.1)	
**Qualifications (attained or studying towards) [c]**				
No academic qualifications			1 (0.27)	
GCSES (or equivalent level)			13 (3.6)	
AS/A Levels (or equivalent level)			34 (9.3)	
Higher education qualifications (or equivalent level)			316 (86.5)	
Not sure or other			1 (0.27)	
**Employment status [c]**				
Employed			265 (72.6)	
Parent/carer			2 (0.6)	
Student			93 (25.5)	
Unemployed			5 (1.3)	
**Contraceptive history and use**				
**Used oral contraception before? [c]**				
Yes			352 (96.4)	
No			13 (3.6)	
**Taken combined oral contraceptive (COC) before? [c]**				
Yes	779 (93.3)	428 (91.1)	351 (96.2)	0.003 [d]
No	56 (6.7)	42 (8.9)	14 (3.8)	
**Reason for taking the pill? [c]**				
To prevent pregnancy			302 (82.7)	
For other reasons			40 (11.0)	
To both prevent pregnancy and for other reasons			23 (6.3)	
**Online service use**				
**Ordered COC from online service before? [a]**				
Yes			94 (25.7)	
No			271 (74.3)	
**Prescribed COC from online service?**				
Yes	726 (86.9)	405 (86.2)	321 (87.9)	0.45 [d]
No	109 (13.1)	65 (13.8)	44 (12.1)	

[a] Reported by user at time of order with the online service
[b] Index of multiple deprivation data unavailable for 11 users
[c] Self-reported by participant at research visit, otherwise not collected by the online provider
[d] To compare demographic information, pill and service use between eligible users who did and did not participate in the study, t- and chi-squared tests were carried out. P values are given to 1 dp

Participants had a mean age of 24 years, the majority (221/365, 60.6%) were White British and 86.6% (316/365) had higher educational qualifications. Almost all (351/365, 96.2%) had used COC before, 74.3% (271/365) had not ordered COC online before, and 87.9% (321/ 365) were prescribed the COC after a full assessment of medical eligibility. Eligible users who participated in the study

For peer review only

For peer review only - http://bmjopen.bmj.com/site/about/guidelines.xhtml

were similar to those who did not although the proportion of new users was higher among non-participants (table 1).

3.2. Strategies used to obtain measurements

The majority of participants (93.4%, 341/365) reported feeling confident self-reporting height, weight and BP, while 6.6% (24/365) did not. Of 365 participants, 327 provided data on their process of BP measurement that made classification of clinical appropriateness possible, and 90.5% of these (296/327) reported processes that were clinically appropriate (supplementary material A).

Half of participants (50.1%, 181/361) measured their weight at home, 31.0% (112/361) reported a measurement from a clinic, 3.3% (12/361) from a pharmacy, 14.7% (53/361) from non-clinical setting, such workplace, gym or university, and 0.8% (3/361) participants reported estimating their weight measurements.

3.3. Agreement between BP and BMI readings

Based on researcher- measured figures, the prevalence of UKMEC 3&4 BMI and BP was 1.4% (5/356) and 3.0% (11/365) respectively. Kappa statistics suggest that the agreement between self-reported and researcher-measured BMI data was substantial (0.72 [95% confidence intervals [CI] 0.42, 1.00]), but agreement between self-reported and researcher-measured BP was poor (0.06 [95% CI -0.11, 0.23]). Two-by-two tables from which agreement was derived are presented in supplementary material B and C, full results are presented in table 2. Self-reported height and weight readings identified 80.0% (95% CI 28.4 – 99.5) of individuals with a researcher-measured BMI corresponding to UKMEC 3 or 4. Self-reported BP readings identified 9.1% (95% CI 28.4 – 99.5) of those with a researcher-measured BP corresponding to UKMEC 3 or 4. The specificity of self-reported BMI and BP measurements was high at 99.4% (95% CI 98.0- 99.9), and 97.2% (95% CI 94.9 – 98.6) respectively. Of the ten participants who were found to have self-reported BP UKMEC 1 or 2, but measured as a 3 or 4, eight reported clinically appropriate BP measurement processes and two reported unclassifiable processes.

Table 2: Agreement between self-reported and researcher-measured values

For peer review only - http://bmjopen.bmj.com/site/about/guidelines.xhtml

	N	Prevalence of UKMEC 3&4 (n, %)	Sensitivity % (95% CI)	Specificity % (95% CI)	Positive predictive value % (95% CI)	Negative predictive value % (95% CI)	Cohen's Kappa coefficient	
							Kappa coefficient (95% CI) *	P value
**UKMEC categorisation for BMI (1&2 vs 3&4)**	365	5 (1.4%)	80.0 (28.4, 99.5) %	99.4 (98.0, 99.9) %	66.7 (22.3, 95.7) %	99.7 (98.5, 100.00) %	0.72 (0.42, 1.00)	0.0000
**Blood pressure (<140/90 vs > or= 140/90)**	365	11 (3.0%)	9.1 (0.23, 41.3) %	97.2 (94.9, 98.6) %	9.1 (0.23, 41.3) %	97.2 (94.9, 98.6) %	0.06 (-0.11, 0.23)	0.1156

216 Self-reported weight was on average 1.8 kg lower (95% CI -2.4, -1.2) and the resulting BMI on average 0.70 kg/cm[2] lower (95% CI -0.94, -0.47) than researcher-measured readings. Systolic BP was 8mmHg higher (95% CI 7.1, 9.6) in the self-reported data than researcher-measured data. Average differences for height were <1mm (95% CI 0.0002, 0.008) and for diastolic BP were <1mmHg (95% CI -0.54, 1.44) (see table 3).

Table 3: Mean differences between self-reported and measured values; Pearson's correlation coefficient and Bland Altman limits of agreement

	Mean (SD)		Mean difference (95% CI); SD	Pearson's correlation coefficient	Bland Altman limits of agreement
	Self-reported values	Measured values			
**Height (m)**	1.65 (0.07)	1.65 (0.06)	0.004 (0.000, 0.008); 0.038	0.844	-
**Weight (kg)**	62.7 (10.4)	64.5 (12.4)	-1.8 [a] (-2.4, -1.2); 5.8	0.883	-
**Body mass index (kg/m2)**	22.9 (3.6)	23.6 (4.1)	-0.70 [a] (-0.94, -0.47); 2.3	0.832	- 5.2, 3.8
**Systolic blood pressure (mmHg)**	115.8 (10.4)	107.5 (9.7)	8.36 [a] (7.1, 9.6); 11.9	0.300	-15.4 to 32.1
**Diastolic blood pressure (mmHg)**	74.8 (8.5)	74.3 (7.4)	0.45 (-0.54, 1.4); 9.6	0.276	-18.8, 19.7

[a] $P < 0.05$, paired t test, difference between self-reported and measured values versus Ho: mean(diff) =0

Bland Altman plots (figures 2-4) for BMI and systolic and diastolic BP illustrate the degree of agreement between self-reported and researched-measured values (17). Normal distribution of the differences was verified with a histogram. Limits of agreement are wider in systolic and diastolic BP than in BMI (also see table 3). The plots show that for both BP and BMI there was no trend in differences between measurements; participants with both a high and low mean BP or BMI from self-reported and researcher measurements, were found to have variable differences in self-reported and measured BP or BMI.

4. Discussion

4.1. Key findings

For peer review only - http://bmjopen.bmj.com/site/about/guidelines.xhtml

Self-reported BMI was found to have substantial agreement with researcher-measured BMI, unlike self-reported BP, which had poor agreement. The majority of participants undertook a clinically acceptable process in obtaining self-reported BP. On average, participants slightly under-reported their weight and over-reported their systolic BP. Bland Altman plots showed better agreement for BMI than for systolic and diastolic BP. The plots also showed no trends or systemic variation in differences between self-reported and researcher measurements.

4.2. Where this sits within the literature

Research from Australia, Hong Kong, Finland, Malaysia, Sweden and the United States has found similar, substantial agreement for self-reported and researcher/clinician-measured weight and height for populations of all ages (18–23). Similar to our findings, in other studies which compared both self-reported and researcher-measured BP and BMI, agreement was stronger between BMI measures than between BP measurements (21,24). Studies comparing self-reported and researcher/clinician-measured BP have found varying levels of agreement, with higher agreement often found older populations (24–26). A large cohort study (n=1537) in New Zealand found kappa between self-reported and measured BP in 18-44 year olds to be 0.26 (p <0.001), with a sensitivity of 18.1% and a specificity of 99.1% (25). Within the same study, those aged 45-64 had an agreement of 0.49 (p <0.001), a sensitivity of 47.5%. A smaller study (n=200) of Italian women in their 50s found a kappa of 0.319 for self-reported compared with measured hypertension (24). Self-measurement of BP is more accurate than clinician-measured BP for those who are hypertensive, at risk of hypertension or are pregnant, possibly because they receive support and training for correct measurement (27–29). Self-monitoring of BP is becoming a common activity for some of these patient groups, for example amongst both hypertensive and non-hypertensive pregnant women, and those who self-monitor may have better knowledge about their BP (30).

Findings from a nested qualitative sample within this study reflect a difference in self-reporting BMI and BP experiences, where height and weight measurements were accessible and familiar but BP readings were not. The qualitative study showed that participants worked hard to understand and measure BP through a combination of recent/past measurements, borrowed machines, health service visits and online research. This analysis suggests the importance of the acknowledgement of work required to measure BP, evidence of credible human support and a digital interface that communicates the health benefits of accurate measurement (15).

4.3. Strengths and limitations

For peer review only - http://bmjopen.bmj.com/site/about/guidelines.xhtml

This is the first study of its kind to observe self-reporting of BP and BMI to obtain the COC in a young, healthy population of reproductive age. It is timely as innovative, self-care interventions for SRH become more prevalent (31,32).

We have compared self-reported and researcher-measured BP. Although, prior to contraception provision, clinicians taking single measures of blood pressure is standard, the assumption that the measurement from the research visit reflects the true BP is problematic. BP is inherently variable, influenced by numerous extrinsic and intrinsic factors (33). A systematic review of the sources of inaccuracy in the measurement of adult resting BP, identified significant directional effects from 27 sources, resulting in a range of -23.6 to +33.0 mmHg for systolic BP and -14.0 to +23.0 mmHg for diastolic BP (34). Whilst researchers employed recommended strategies to mitigate inaccuracies in BP measurement, some factors remain irremediable, for example 'white coat syndrome', whereby researcher-, or clinician-, measured BP are artificially raised due to anxiety, or natural BP variation (35,36). Recently updated UK guidance recommends ambulatory or home BP monitoring involving multiple repeated measures over a day for ambulatory or at least 4 days for home BP monitoring to diagnose hypertension (13).

There was potential for self-selection bias during recruitment where users who may have estimated their BP during their order might have been unwilling to participate. To mitigate this, invitation communication, whilst providing sufficient information on the study procedure, did not emphasise validation of measurements (see supplementary material D). The study had good uptake, with 44.1% of eligible users attending a research visit, where researchers reiterated their independence from the online service.

Almost all study participants had experience of taking COC before ordering from the online service (96.2%, 351/365), a figure that is greater than amongst non-responders (91.1%, 428/470, p<0.01). This suggests potential healthy user bias, in that those with more experience of COC may be more likely to agree to participate. Such experienced COC users may be less likely to have high BP or BMI, and could be more familiar with, or more aware of the importance of, strategies to obtain biometric readings for remote prescription that might be clinically appropriate.

The medium time between COC order and research visit was 20 days. This may have impacted on participants' recall of the details of recent BP measurements. This is evident in the 38 unclassifiable participant processes: 31 participants gave the date of their most recent BP measurement between their order and visit, seven were unclassifiable due to limited information in their response.

For peer review only

For peer review only - http://bmjopen.bmj.com/site/about/guidelines.xhtml

There are methodological limitations that may explain the low kappa coefficient. The kappa produced, in particular for BP, should be interpreted considering the method's paradoxes and problems. The two-by-two table for BP (supplementary material C) produced, with it's perfectly symmetrically unbalanced marginal totals, satisfies both paradoxes outlined by Feinstein and Cicchetti, which, in addition to the low prevalence of researcher-measured high BMI and BP, perhaps provides an additional factor explaining the low kappa in light of high percentage agreement (37).

4.4. Meaning and mechanism

Explanations for our findings include the natural variability of BP, white coat syndrome, or inaccurate self-measurement or reporting (35,36). Although our quantitative data does not allow us to explore whether our findings are due to the natural variability of BP or to inaccurate self-measurement or reporting, our qualitative research provides some insights (15). Participants shared both experiences of white coat syndrome at previous consultations, and, in relation to their online COC order, difficulties obtaining a new BP measurement, remembering values from a recent reading, and difficulties interpreting or uploading readings, as well as a single participant who recounted misreporting despite a known high BP.

Whilst BP limits of agreement are comparable to biologically plausible ranges established when estimating the impact of sources of inaccuracy (34), the clinically relevant factor for remote COC prescription is not the magnitude of these limits, but whether they shift BP from a non-contraindication to a contraindication. Nevertheless, at their widest, such limits would trigger clinical concern and might initiate a conversation about process and accuracy of measurement.

The difference in findings for BP and BMI might reflect differences in familiarity with their measurement. Fifty years ago, accurate equipment for measuring weight were rare in households and most people did not know their current weight. BP monitors are now transitioning from a technology predominantly available in health care settings to one that is increasingly available at home. Strategies to increase accuracy of reporting could focus on improving understanding of BP and access to measurement.

5. Implications and recommendations for online contraceptive services

This study's key findings, understood in the context of findings from the nested qualitative study (15), have implications for both online and face-to-face contraceptive services. In order to maximise the benefits and minimise the risks of the online provision of COC, a self-care intervention, service providers need to take an active role in creating a 'safe and supportive enabling environment' for

For peer review only - http://bmjopen.bmj.com/site/about/guidelines.xhtml

those seeking to obtain an online COC prescription. This includes acknowledging their responsibility in supporting users to self-report accurate biometric readings (31,38).

In response to the findings, in collaboration with the study team, SH:24, the service evaluated in this study, re-designed their online COC clinical pathway. This was completed through a process of user-centred design. The design process suggested innovations that include: building skills for self-monitoring in face-to-face care when BP is measured by clinicians; providing home BP monitors with support to use them; improved online information on the importance of BP measurements and consequences of inaccurate reporting; credible clinical support as required; issuing a short supply for the first COC order with information on how to obtain a BP measurement (15,39). These strategies may improve the accuracy of BP reporting and we recommend further research to evaluate them.

6. Conclusions

Online contraceptive services provide a convenient alternative for oral contraception users which may improve uptake and continuation (5,40). Accuracy of self-reported height, weight and BP is important to ensure appropriate, safe COC prescription online, since BMI and BP may contraindicate use (6,7).

Whilst self-reported BMI was found to have substantial agreement with researcher-measured BMI, self-reported BP was shown to have poor agreement with researcher-measured BP. These differences may be due to the natural variability of BP, white coat syndrome, methodological limitations associated with kappa or by inaccurate self-measurement and reporting.

As online contraceptive services are increasingly prevalent and self-measured BP has been shown to be valid in other populations, strategies to improve BP self-measurement for remote prescription of COC are needed.

<u>Acknowledgements</u>

We thank all users of the online service who took part in this study. We thank SH:24 for collaborating on the project, in particular the clinical team, designers and data analyst at the online service for their support with recruitment and data collection.

<u>Ethical considerations</u>

The East Midlands – Leicester Research Ethics Committee granted ethical approval to the project (Evaluation of online provision of oral contraceptives to measure: accuracy of self-reported height, weight and blood pressure; essential information transfer and user experience, reference 17/EM/0181) on 6th June 2017. Before participating in a research visit, users were presented with a

For peer review only - http://bmjopen.bmj.com/site/about/guidelines.xhtml

For peer review only

participant information sheet, were given the opportunity to ask questions, which they had answered to their satisfaction, and then provided informed consent. Participation was voluntary, and participants were assured that the information they provided would be anonymised and kept confidential. All participants whose biometric data suggested a UKMEC category 3 or 4 or who reported contraindications to the COC during the study or indicated a need for further clinical support were referred to a local sexual health service.

Funding

This report is independent research funded by the National Institute for Health Research (Research for Patient Benefit Programme, PB-PG-0815-20009). The views expressed in this publication are those of the author(s) and not necessarily those of the NIHR or the Department for Health and Social Care. The sponsor, King's College Hospital, was not involved in study design, data collection, analysis, or interpretation of data.

Data statement

The anonymised dataset is available from the authors on request.

Competing interests

Since completion of this study, HM has worked at the online service on a different research project. PB is clinical director of the online service studied and is one of the prescribers within this service. AM has also been employed by the online service as a bank clinical support midwife. This work is part of a process of research on innovation at SH:24 where all innovation is the subject of research to ensure that learning is shared widely. SH:24 is a 'not for profit' organisation that provides health services to the National Health Service and sharing learning through innovation and research is one of the principles of the organisation.

Author contributions

Hannah McCulloch contributed to data collection, led the data analysis, and drafted the manuscript.

Alessandra Morelli contributed to data collection and data analysis and commented on the manuscript.

Caroline Free contributed to the conception and design of the study, contributed to data analysis and commented on the manuscript.

Jonathan Syred contributed to the conception and design of the study, contributed to data collection and commented on the manuscript.

For peer review only

Riley Botelle contributed to data collection and commented on the manuscript.

Paula Baraitser contributed to the conception and design of the study, oversaw data collection, contributed to data analysis and commented on the manuscript.

Figure headings

Figure 1: Recruitment Flow Chart for users of the online service

Figure 2. Bland Altman plot of the differences between self-reported and researcher-measured BMI compared with the mean self-reported and researcher-measured BMI. The central line represents the mean difference between self-reported and researcher-measured BMI. The upper and lower boundaries represent the 95% limits of agreement.

Figure 3: Bland Altman plot of the differences between self-reported and researcher-measured systolic BP compared with the mean self-reported and researcher-measured systolic BP. The central line represents the mean difference between self-reported and researcher-measured systolic BP. The upper and lower boundaries represent the 95% limits of agreement.

Figure 4. Bland Altman plot of the differences between self-reported and researcher-measured diastolic BP compared with the mean self-reported and researcher-measured diastolic BP. The central line represents the mean difference between self-reported and researcher-measured diastolic BP. The upper and lower boundaries represent the 95% limits of agreement.

References

1. French RS, Gibson L, Geary R, Glasier A, Wellings K. Changes in the prevalence and profile of users of contraception in Britain 2000–2010: evidence from two National Surveys of Sexual Attitudes and Lifestyles. BMJ Sexual & Reproductive Health [Internet]. 2020 Jan 21 [cited 2020 Jan 29];bmjsrh-2019-200474. Available from: http://jfprhc.bmj.com/lookup/doi/10.1136/bmjsrh-2019-200474

2. Royal College of Obstetricians and Gynaecologists. Better for Women: Improving the health and wellbeing of girls and women [Internet]. 2019. Available from: https://www.rcog.org.uk/en/news/campaigns-and-opinions/better-for-women/

3. Wilson E, Baraitser P, Free C. Does internet-accessed STI (e-STI) testing increase testing uptake for chlamydia and other STIs among a young population who have never tested? Secondary analyses of data from a randomised controlled trial. Sex Transm Infect [Internet]. 2019 [cited 2019 Jul 15];0:1–6. Available from: http://sti.bmj.com/

4. Wilson E, Free C, Morris T, Syred J, Ahamed I, Menon-Johansson A, et al. Internet-accessed sexually transmitted infection (e-STI) testing and results service: A randomised, single-blind, controlled trial. PLoS ONE. 2017;14(12).

For peer review only

For peer review only - http://bmjopen.bmj.com/site/about/guidelines.xhtml

5. Rezel-Potts E, Free C, Syred J, Baraitser P. Expanding choice through online contraception: A theory of change to inform service development and evaluation. BMJ Sexual and Reproductive Health. 2019;

6. Faculty of Sexual and Reproductive Healthcare. UK Medical eligibility criteria for contraceptive use: UKMEC 2016 (Updated 2019).

7. World Health Organisation. Medical Eligibility Criteria for Contraceptive Use [Internet]. 2015. Available from: https://www.who.int/reproductivehealth/publications/family_planning/Ex-Summ-MEC-5/en/

8. SH:24. Programme overview [Internet]. Available from: https://sh24.org.uk/

9. Public Health England. Sexual and Reproductive Health Profiles [Internet]. 2020 [cited 2020 Mar 4]. Available from: https://fingertips.phe.org.uk/profile/sexualhealth/data#page/0/gid/8000057/pat/6/par/E12000007/ati/102/are/E09000028

10. Lambeth Southwark and Lewisham Public Health Departments. Lambeth, Southwark and Lewisham Sexual and Reproductive Health Strategy 2019–24 [Internet]. London; 2019. Available from: https://www.lambeth.gov.uk/sites/default/files/yh-lsl-sexual-health-strategy_0.pdf

11. General Medical Council. Good practice in prescribing and managing medicines and devices [Internet]. 2013. Available from: https://www.gmc-uk.org/Prescribing_guidance.pdf_59055247.pdf

12. Topouchian J, Agnoletti D, Blacher J, Youssef A, Chahine MN, Ibanez I, et al. Validation of four devices: Omron M6 Comfort, Omron HEM-7420, Withings BP-800, and Polygreen KP-7670 for home blood pressure measurement according to the European society of hypertension international protocol. Vascular Health and Risk Management. 2014;10:33–44.

13. National Institute for Health and Care Excellence. Hypertension in adults: diagnosis and management [Internet]. Available from: https://www.nice.org.uk/guidance/ng136

14. Sim J, Wright CC. The Kappa Statistic in Reliability Studies: Use, Interpretation, and Sample Size Requirements. Physical Therapy [Internet]. 2005 Mar 1 [cited 2020 Jan 31];85(3):257–68. Available from: https://academic.oup.com/ptj/article/85/3/257/2805022

15. Baraitser P, McCulloch H, Morelli A, Free C. How do users of a digital-only contraceptive service provide biometric measurements and what does this teach us about safe and effective online care? A qualitative interview study. BMJ Open. 2020;10(9).

16. Faculty of Sexual & Reproductive Healthcare (FSRH). Collaboration, Choice, Care: The Contraception Priority Setting Partnership (PSP) [Internet]. 2018 [cited 2021 Jun 29]. Available from: https://www.fsrh.org/documents/fsrh-contraception-psp-report-2018-jla/

17. Giavarina D. Understanding Bland Altman analysis. Biochemia Medica. 2015;25(2):141–51.

18. Davies A, Wellard-Cole L, Rangan A, Allman-Farinelli M. Validity of self-reported weight and height for BMI classification: A cross-sectional study among young adults. Nutrition. 2020 Mar 1;71:110622.

For peer review only - http://bmjopen.bmj.com/site/about/guidelines.xhtml

19. Dahl A, Hassing L, Fransson E, Pederson N. Agreement between self-reported and measured height, weight and body mass index in old age—a longitudinal study with 20 years of follow-up. Age and Ageing [Internet]. 2010 [cited 2020 Feb 27];39(4):445–51. Available from: https://www.ncbi.nlm.nih.gov/pmc/articles/PMC2899942/

20. Tuomela J, Kaprio J, Sipilä PN, Silventoinen K, Wang X, Ollikainen M, et al. Accuracy of self-reported anthropometric measures — Findings from the Finnish Twin Study. Obesity Research and Clinical Practice. 2019 Nov 1;13(6):522–8.

21. Murphy G, Snetselaar L, Myers E, LaForge B, Qualls LG, Hand RK, et al. Self-reported health parameters compared with clinician measurements: methods in practice-based research. J Public Health Manag Pract [Internet]. 2014 [cited 2022 Jan 10];20(5):513–22. Available from: https://pubmed.ncbi.nlm.nih.gov/24352004/

22. Olfert MD, Barr ML, Charlier CM, Famodu OA, Zhou W, Mathews AE, et al. Self-Reported vs. Measured Height, Weight, and BMI in Young Adults. Int J Environ Res Public Health [Internet]. 2018 Oct 11 [cited 2022 Jan 10];15(10). Available from: https://pubmed.ncbi.nlm.nih.gov/30314261/

23. Kee CC, Lim KH, Sumarni MG, Teh CH, Chan YY, Nuur Hafizah MI, et al. Validity of self-reported weight and height: a cross-sectional study among Malaysian adolescents. BMC Med Res Methodol [Internet]. 2017 Jun 2 [cited 2022 Jan 10];17(1). Available from: https://pubmed.ncbi.nlm.nih.gov/28577547/

24. Villarini M, Acito M, Gianfredi V, Berrino F, Gargano G, Somaini M, et al. Validation of Self-Reported Anthropometric Measures and Body Mass Index in a Subcohort of the DianaWeb Population Study. Clinical Breast Cancer [Internet]. 2019 Aug 1 [cited 2020 Feb 20];19(4):e511–8. Available from: http://www.ncbi.nlm.nih.gov/pubmed/31182401

25. Taylor A, Grande ED, Gill T, Pickering S, Grant J, Adams R, et al. Comparing self-reported and measured high blood pressure and high cholesterol status using data from a large representative cohort study. Australian and New Zealand Journal of Public Health [Internet]. 2010 Aug 1 [cited 2020 Feb 20];34(4):394–400. Available from: http://doi.wiley.com/10.1111/j.1753-6405.2010.00572.x

26. Ning M, Zhang Q, Yang M. Comparison of self-reported and biomedical data on hypertension and diabetes: Findings from the China Health and Retirement Longitudinal Study (CHARLS). BMJ Open. 2016;6(1).

27. McManus RJ, Wood S, Bray EP, Glasziou P, Hayen A, Heneghan C, et al. Self-monitoring in hypertension: A web-based survey of primary care physicians. Journal of Human Hypertension. 2014;28(2):123–7.

28. Hinton L, Tucker KL, Greenfield SM, Hodgkinson JA, Mackillop L, McCourt C, et al. Blood pressure self-monitoring in pregnancy (BuMP) feasibility study; A qualitative analysis of women's experiences of self-monitoring. BMC Pregnancy and Childbirth. 2017 Dec 19;17(1).

29. Tucker KL, Taylor KS, Crawford C, Hodgkinson JA, Bankhead C, Carver T, et al. Blood pressure self-monitoring in pregnancy: Examining feasibility in a prospective cohort study. BMC Pregnancy and Childbirth. 2017 Dec 28;17(1).

30. Tucker KL, Hodgkinson J, Wilson HM, Crawford C, Stevens R, Lay-Flurrie S, et al. Current prevalence of self-monitoring of blood pressure during pregnancy: the BUMP Survey. J

For peer review only - http://bmjopen.bmj.com/site/about/guidelines.xhtml

Hypertens [Internet]. 2021 May 1 [cited 2022 Jan 10];39(5):994–1001. Available from: https://pubmed.ncbi.nlm.nih.gov/33399304/

31. Narasimhan M, Allotey P, Hardon A. Self care interventions to advance health and wellbeing: A conceptual framework to inform normative guidance. BMJ (Online). 2019;365.

32. World Health Organization (WHO). WHO Consolidated Guideline on Self-Care Interventions for Health: Sexual and Reproductive Health and Rights. 2020; Available from: https://apps.who.int/iris/bitstream/handle/10665/325480/9789241550550-eng.pdf?ua=1

33. Pickering TG. Blood pressure variability and ambulatory monitoring. [Internet]. Vol. 2, Current opinion in nephrology and hypertension. 1993 [cited 2020 Mar 2]. p. 380–5. Available from: http://www.ncbi.nlm.nih.gov/pubmed/7922198

34. Kallioinen N, Hill A, Horswill MS, Ward HE, Watson MO. Sources of inaccuracy in the measurement of adult patients' resting blood pressure in clinical settings: A systematic review. Vol. 35, Journal of Hypertension. Lippincott Williams and Wilkins; 2017. p. 421–41.

35. McGrath BP. Ambulatory blood pressure monitoring and "white coat" hypertension: Saving costs. Vol. 176, Medical Journal of Australia. 2002. p. 571–2.

36. Turner MJ, van Schalkwyk JM. Blood pressure variability causes spurious identification of hypertension in clinical studies: A computer simulation study. American Journal of Hypertension. 2008 Jan;21(1):85–91.

37. Feinstein AR, Cicchetti D v. High agreement but low Kappa: I. the problems of two paradoxes. Journal of Clinical Epidemiology. 1990;43(6):543–9.

38. Ferguson L, Fried S, Matsaseng T, Ravindran S, Gruskin S. Human rights and legal dimensions of self care interventions for sexual and reproductive health. The BMJ. 2019;365.

39. Baraitser P, McCulloch H, Shather Z, Free C. Supporting blood pressure measurement prior to online prescription of the combined oral contraceptive. BMJ Sexual and Reproductive Health. 2021;

40. Rezel-Potts E, Palmer MJ, Free C, Baraitser P. A cohort study of the service-users of online contraception. BMJ Sexual and Reproductive Health. 2020;46(4):287–93.

For peer review only

For peer review only - http://bmjopen.bmj.com/site/about/guidelines.xhtml

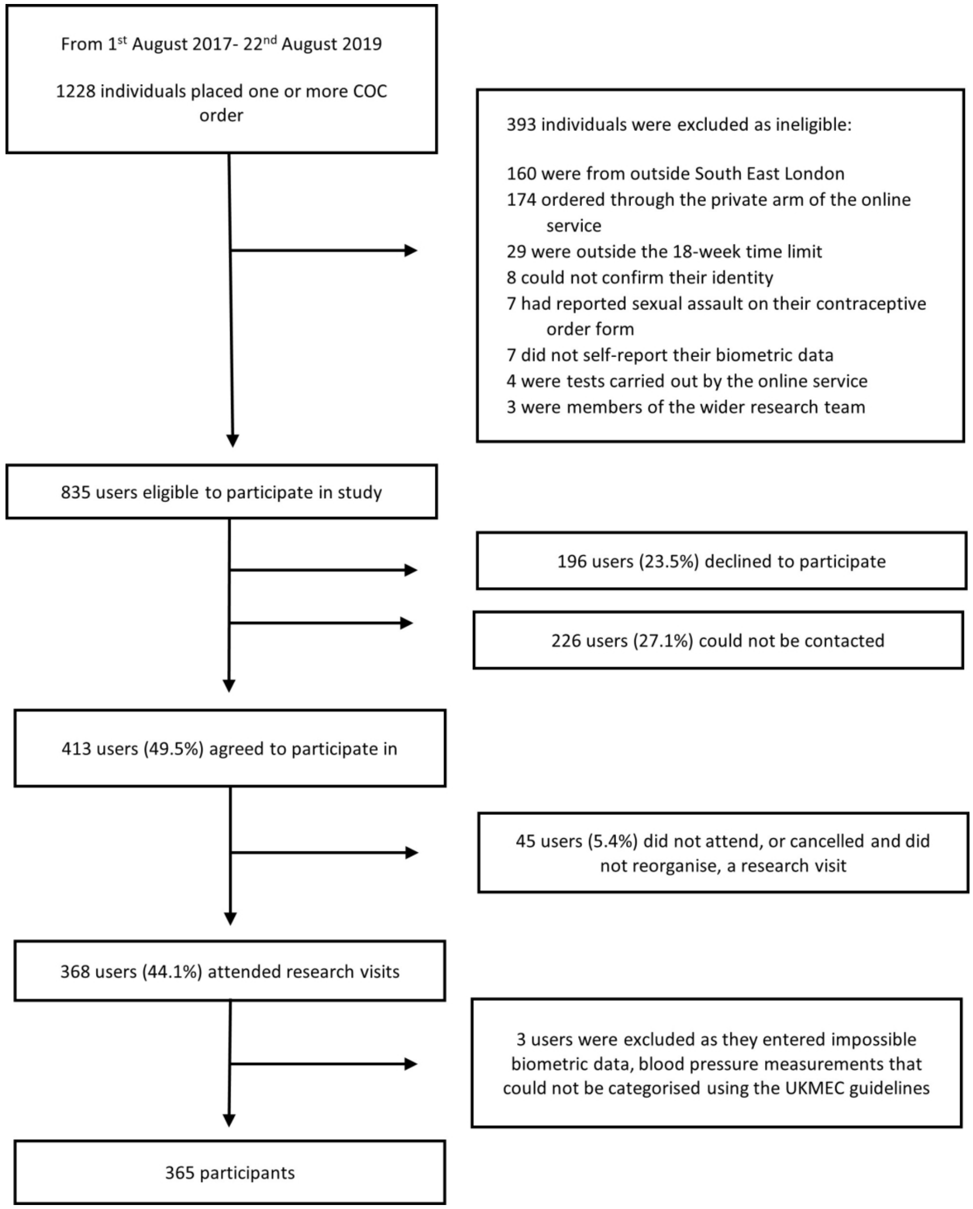

Figure 1: Recruitment Flow Chart for users of the online service

154x192mm (330 x 330 DPI)

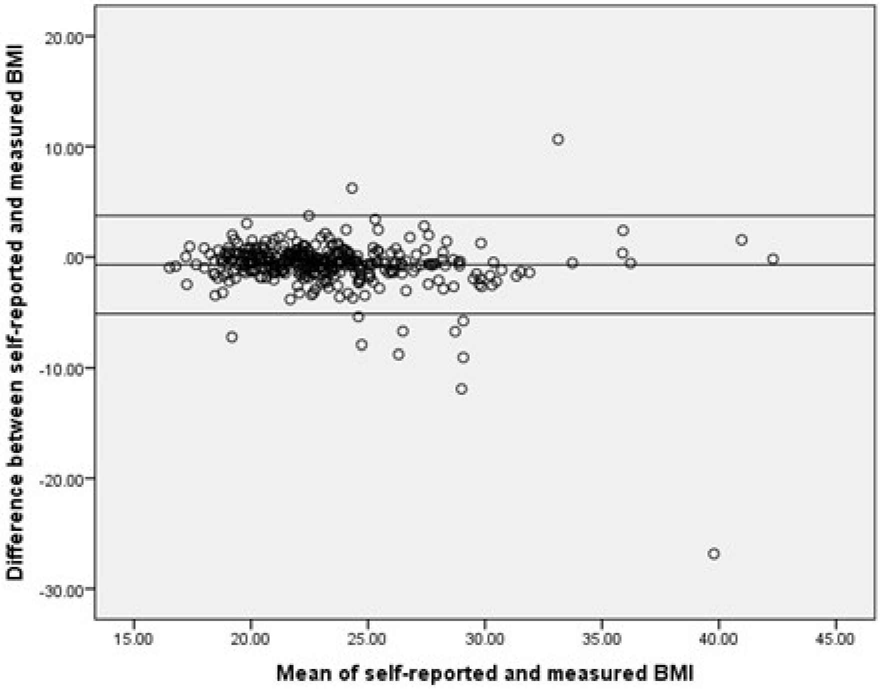

Figure 2. Bland Altman plot of the differences between self-reported and researcher-measured BMI compared with the mean self-reported and researcher-measured BMI. The central line represents the mean difference between self-reported and researcher-measured BMI. The upper and lower boundaries represent the 95% limits of agreement.

119x93mm (96 x 96 DPI)

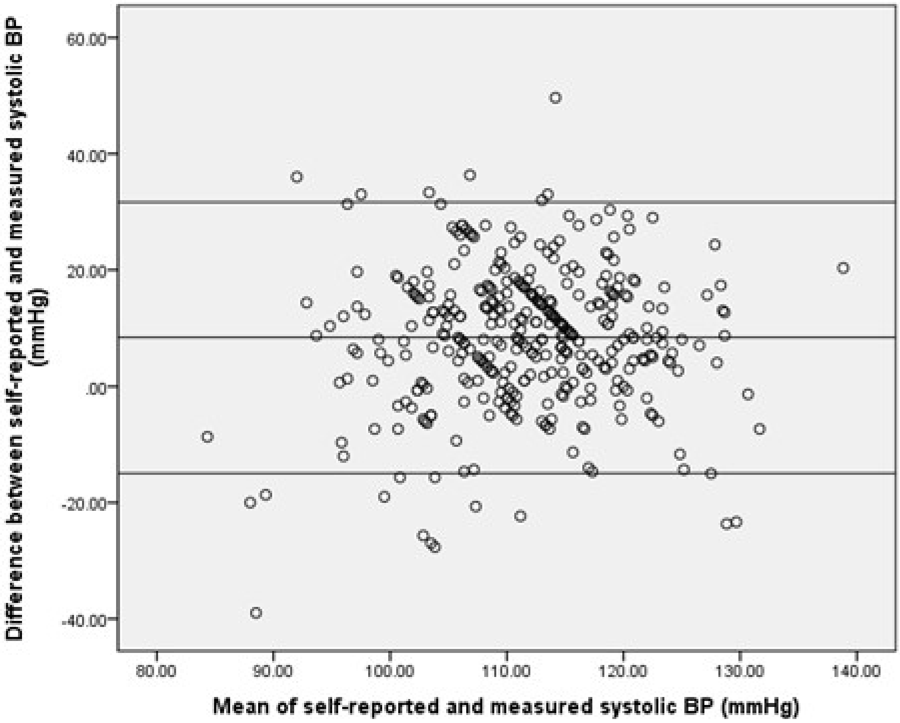

Figure 3: Bland Altman plot of the differences between self-reported and researcher-measured systolic BP compared with the mean self-reported and researcher-measured systolic BP. The central line represents the mean difference between self-reported and researcher-measured systolic BP. The upper and lower boundaries represent the 95% limits of agreement

122x97mm (96 x 96 DPI)

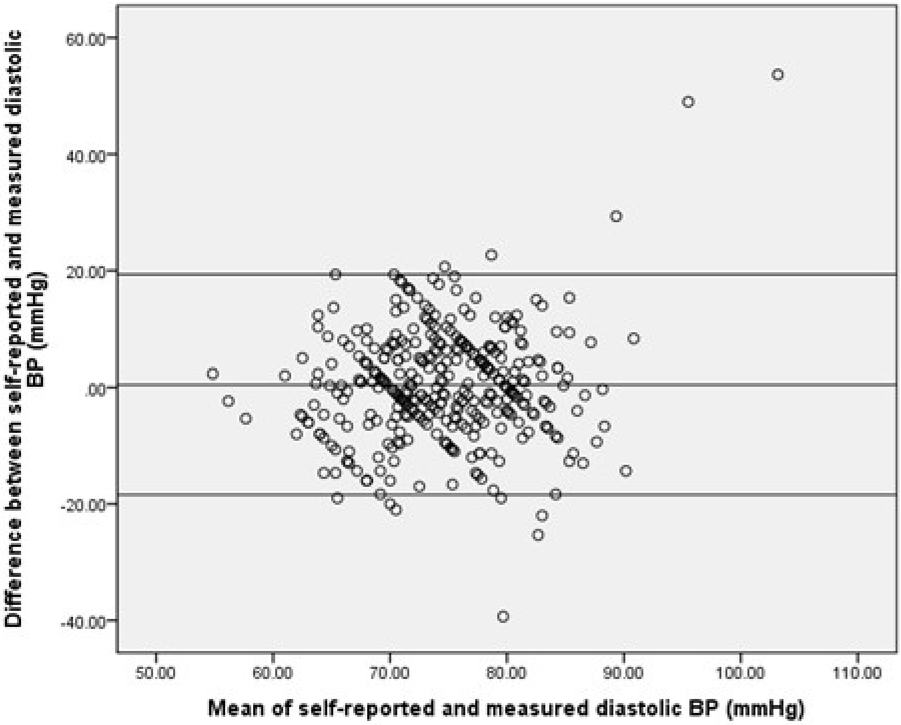

Figure 4. Bland Altman plot of the differences between self-reported and researcher-measured diastolic BP compared with the mean self-reported and researcher-measured diastolic BP. The central line represents the mean difference between self-reported and researcher-measured diastolic BP. The upper and lower boundaries represent the 95% limits of agreement.

122x98mm (96 x 96 DPI)

Supplementary material

A. Process of BP measurement and examples from free text answers

Process of blood pressure measurement	Example
Unclassifiable	- I have a blood pressure monitor at home (date given between order and research visit)<br>- "My blood pressure was measured by a colleague at work (12 days before order)<br>- "[South East London Clinic] (date given between order and research visit)"<br>- "I have a nurse at my office that offer blood pressure testing so I used the facilities provided to check (date given between order and research visit)"<br>- "I visited the pharmacy stand in boots. They took me to a private room and took my blood pressure for me (date given between order and research visit)"
Clinically appropriate	- "From my GP when I was first prescribed the pill (3 months before order)"<br>- "The nurse in the clinic took my blood pressure (same day as order)"<br>- "Inherited blood pressure machine from relative with long term illness who no longer needed it. Used this at home to obtain measure (4 days before order)<br>- "I visited my aunts house to get my blood pressure reading since she has a machine at home (day before order)<br>- "I went to my local pharmacy where they had a self checker machine that calculated my blood pressure, weight and height (same day as order)"
Clinically inappropriate	- "At the GP before getting a repeat prescription of the pill, which was measured normally (18 months before order)"<br>- "A bupa nurse came into my place of work and offered a health check. My blood pressure was taken there (2 years before order)<br>- "I looked up what a healthy blood pressure would be on NHS website and put in that blood pressure when on the sh:24 website"<br>- "Estimated. Had a blood pressure machine but feel I did not use it correctly"<br>- "Googled healthy blood pressure"

For peer review only - http://bmjopen.bmj.com/site/about/guidelines.xhtml

B. Self-reported and researcher-measured BMI

		Measured BMI		
		**UKMEC 1&2 <35.0 kg/m**	**UKMEC 3&4 > or= 35 kg/m**	**Total**
**Self-reported BMI**	**UKMEC 1&2 <35.0 kg/m**	358	1	359
	**UKMEC 3&4 > or= 35 kg/m**	2	4	6
	**Total**	360	5	365

C. Self-reported and researcher-measured BP

		Researcher-measured blood pressure (mmHg)		
		**< 140/90**	**> or = 140/90**	**Total**
**Self-reported blood pressure (mmHg)**	**<140/90**	344	10	354
	**> or= 140/90**	10	1	11
	**Total**	354	11	365

D. SMS invitation message

"Hello. SH:24 are working with King's College Hospital to improve contraception services. King's would like to invite you to take part in their research - they will thank you for your time with £45. The research would involve meeting a member of the King's team to allow them to check your height, weight and blood pressure. You will also be asked to complete a short questionnaire (this should take no more than 40 mins in total). Text back NO if you don't want to receive a call back from the King's research team. Thanks, SH:24"

For peer review only
